# Ligand Design and Preparation, Photophysical Properties, and Device Performance of an Encapsulated-Type *Pseudo*-Tris(heteroleptic) Iridium(III) Emitter

**DOI:** 10.1021/acs.inorgchem.2c04106

**Published:** 2023-02-21

**Authors:** Vadim Adamovich, Llorenç Benavent, Pierre-Luc T. Boudreault, Miguel A. Esteruelas, Ana M. López, Enrique Oñate, Jui-Yi Tsai

**Affiliations:** † Departamento de Química Inorgánica, Instituto de Síntesis Química y Catálisis Homogénea (ISQCH), Centro de Innovación en Química Avanzada (ORFEO-CINQA), Universidad de Zaragoza-CSIC, 50009 Zaragoza, Spain; ‡ Universal Display Corporation, Ewing, New Jersey 08618, United States

## Abstract

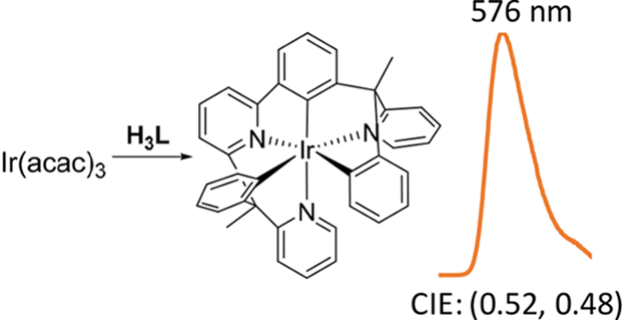

The organic molecule 2-(1-phenyl-1-(pyridin-2-yl)ethyl)-6-(3-(1-phenyl-1-(pyridin-2-yl)ethyl)phenyl)pyridine (**H**_**3**_**L**) has been designed, prepared, and employed to synthesize the encapsulated-type *pseudo*-tris(heteroleptic) iridium(III) derivative Ir(κ^6^-*fac-C,C′,C″-fac-N,N*′*,N″-*L). Its formation takes place as a result of the coordination of the heterocycles to the iridium center and the *ortho*-CH bond activation of the phenyl groups. Dimer [Ir(μ-Cl)(η^4^-COD)]_2_ is suitable for the preparation of this compound of class [Ir(9h)] (9h = 9-electron donor hexadentate ligand), but Ir(acac)_3_ is a more appropriate starting material. Reactions were carried out in 1-phenylethanol. In contrast to the latter, 2-ethoxyethanol promotes the metal carbonylation, inhibiting the full coordination of **H**_**3**_**L**. Complex Ir(κ^6^-*fac-C,C′,C″-fac-N,N*′*,N″-*L) is a phosphorescent emitter upon photoexcitation, which has been employed to fabricate four yellow emitting devices with 1931 CIE (x:y) ∼ (0.52:0.48) and a maximum wavelength at 576 nm. These devices display luminous efficacies, external quantum efficiencies, and power efficacies at 600 cd m^–2^, which lie in the ranges 21.4–31.3 cd A^–1^, 7.8–11.3%, and 10.2–14.1 lm W^1–^, respectively, depending on the device configuration.

## Abstract 2

The molecule 2-(1-phenyl-1-(pyridin-2-yl)ethyl)-6-(3-(1-phenyl-1-(pyridin-2-yl)ethyl)phenyl)pyridine (**H**_**3**_**L**) has been designed, prepared, and employed to synthesize the encapsulated-type *pseudo*-tris(heteroleptic) iridium(III) derivative Ir(κ^6^-*fac-C,C′,C″-fac-N,N*′*,N″-*L), which has been used to fabricate yellow emitting OLED devices.

## Introduction

The OLED devices based on phosphorescent emitters (PHOLEDs) show better performance than those employing fluorescent emissive compounds.^1^ Among the phosphorescent emitters, iridium(III) complexes occupy a prominent position;^2^ their emissions depend on both the metal center and the ligands. As a consequence of ligand dependence, the photophysical properties of emitters of a particular metal can be governed by controlling the arrangement of the donor atoms of its coordination sphere, the reason why the ligand design is of great relevance.^3^ A finer adjustment of the photophysical characteristics is achieved with heteroleptic systems, which can be generated in two alternative ways: by combining different ligands or by mixing different electron donating moieties within the same polydentate ligand. As a consequence of having alternative methodologies, today, the dream of assembling emitters tailored to a certain requirement is closer to being realized.^2,3^

Octahedral iridium(III) compounds bearing different bidentate ligands (b) with the ability of donating 3 electrons, 3b, are of special interest, in particular [3b+3b′+3b″] complexes that coordinate three different 3-electron donor bidentate groups. The reason for the significance of these tris(heteroleptic) compounds is that the presence of three different ligands in the metal allows a finer adjustment of the photophysical characteristics of the emitter. However, they are also difficult to obtain and purify. In addition to having a large number of stereoisomers, these complexes are often involved in redistribution equilibria.^4^ One way to avoid the problem, which arouses interest, is to reduce the number of ligands in the coordination sphere of the metal by increasing the number of donor atoms of some of them. In addition, it is believed that a stronger metal–ligand interaction should increase the efficiency of the emitter, despite the distortions that are generated as a consequence of the greater coordination rigidity.^5^ A first approximation was the use of pincer ligands,^6^ in line with the impact of these groups on transition metal chemistry.^7^ As a consequence, during the past decade a remarkable number of iridium(III) emitters with two different tridentate ligands have been prepared.^8^ In recent years, efforts have been directed to the search for ligands with higher denticity; mainly the nonplanar tetradentates.^9^ Those formed by two different bidentate units (tt′) have been pursued with particular zeal,^10^ although they are still very scarce.

A further step in the development of ligands with high denticity is the design of hexadentates (h). In principle, they should increase the strength of the metal–ligand interaction, decrease the number of stereoisomers, and avoid the formation of mixtures by ligand redistribution. However, their use in organometallics and coordination chemistry is even less frequent than the utilization of tetradentate ones. This class of ligands is of great interest because they allow encapsulating metal ions.^11^ Hexadentate ligands have not been employed in PHOLED technology, although some iridium(III) emitters have been prepared with these groups. They are based on tripodal structures formed by flexible arms attached to the orthometalated phenyl substituent or the heterocycle of three independent and equal units based on 2-phenylpyridine.^12^ These emitters are therefore homoleptic compounds that coordinate a formally hexadentate ligand.

**Chart 1:**
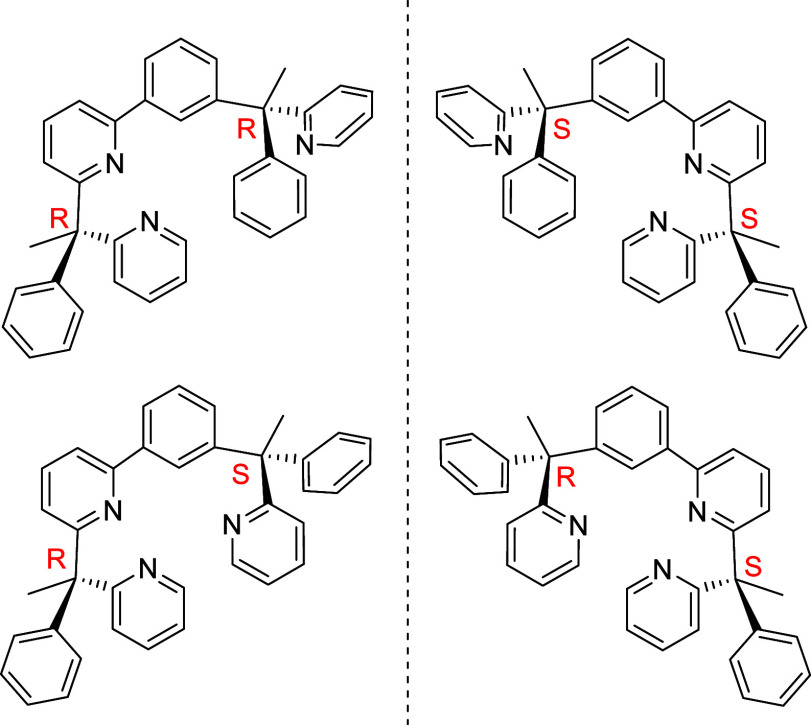
Diastereomers of H_3_L

Our interest in the development of emitters for PHOLED technology^4g,8i,10d,13^ prompted us to design the molecule 2-(1-phenyl-1-(pyridin-2-yl)ethyl)-6-(3-(1-phenyl-1-(pyridin-2-yl)ethyl)phenyl)pyridine (**H**_**3**_**L**), as precursor of the first *pseudo*-tris(heteroleptic) iridium(III) emitter based on a hexadentate ligand. This molecule is formed by a 2-phenylpyridine moiety and two slightly different 2-benzylpyridine units. The difference between the 2-benzylpyridine units is the junction with the 2-phenylpyridine moiety through the respective methylene groups, which act as linkers; a 2-benzylpyridine ties to the phenyl substituent of the 2-phenylpyridine moiety whereas the other connects to the heterocycle. Although subtle, the asymmetry should be enough to promote different contributions of the two 2-benzylpyridine units to the frontier orbitals of the emitter. A nonenantioselective synthesis of this molecule should provide the racemic mixture of the diastereoisomers **H**_**3**_**L**_**RR**_**-H**_**3**_**L**_**SS**_ and **H**_**3**_**L**_**RS**_**-H**_**3**_**L**_**SR**_ shown in Chart 1.

This paper describes the preparation of the designed molecule, its coordination to iridium, the photophysical properties of the resulting complexes, and the first OLED devices based on an emitter bearing a hexadentate ligand.

## Results and Discussion

### Preparation of H_3_L

**Scheme 1:**
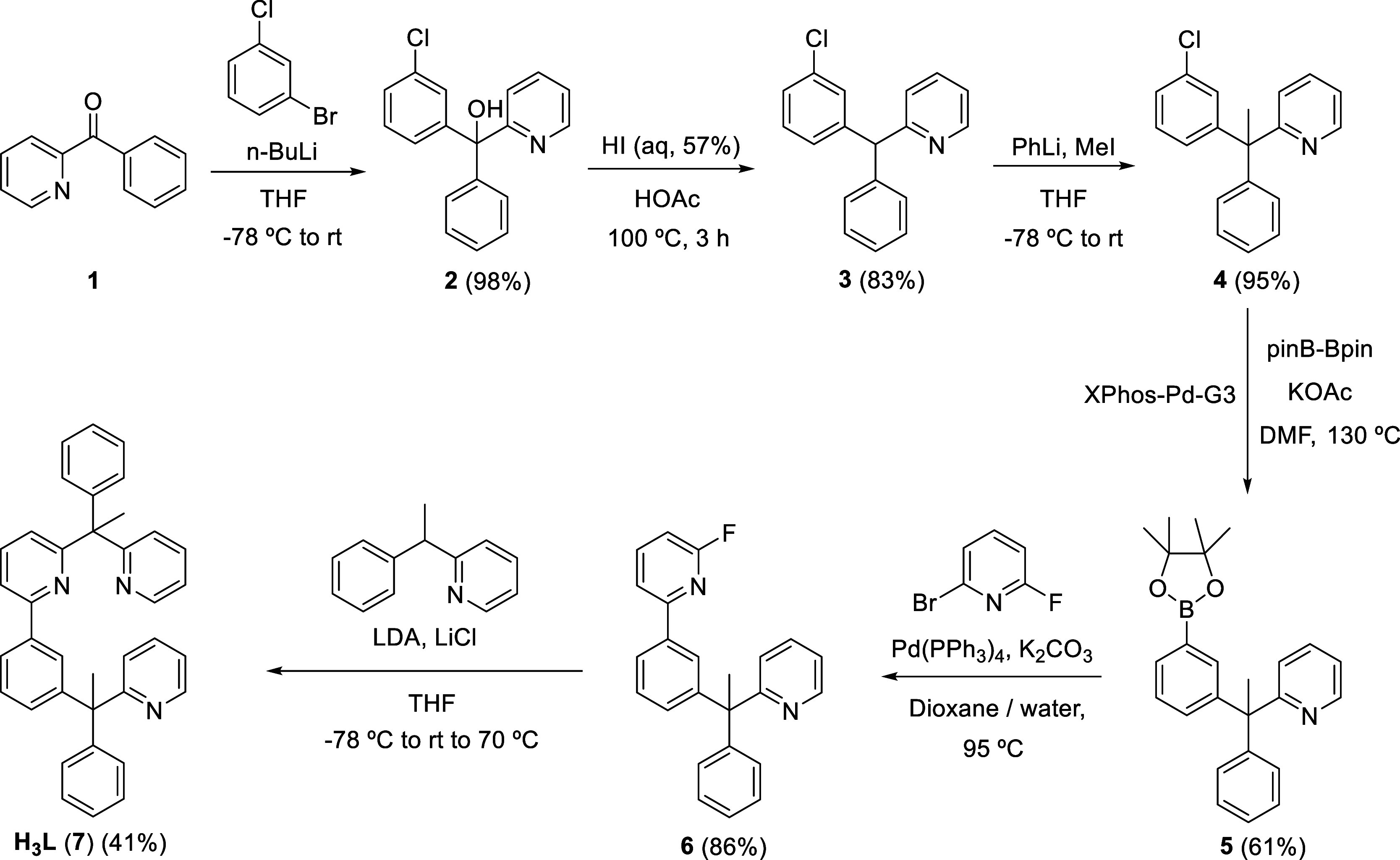
Synthesis of the Proligand H_3_L

The designed molecule was prepared by the procedure shown in Scheme 1. It consisted of six steps, which can be grouped into three stages: introduction of a phenyl fragment into the methylene unit of a 2-benzylpyridine, coupling of said phenyl with a pyridyl group, and linkage of the latter with a second 2-benzylpyridine through its methylene unit.

The first stage is formed by three steps. In the first one, 2-benzoylpyridine (**1**) was used as the synthon for the 2-benzylpyridine that was employed as a support to introduce the phenyl group. The coupling was achieved with an organolithium reagent. The ketone dissolved in tetrahydrofuran was treated with 3-chlorophenyllitium, which was generated *in situ*. The nucleophilic addition of the organometallic reagent to the carbonyl group followed by the hydrolysis of the resulting alcoholate^14^ led to (3-chlorophenyl)(phenyl)(pyridine-2-yl)methanol (**2**), which was isolated as an orange oil in almost quantitative yield. The second step was the direct deoxygenation of the alcohol to form 2-((3-chlorophenyl)(phenyl)methyl)pyridine (**3**), as a brown oil in 83% yield. The reaction was carried out in acetic acid, at 100 °C, using an aqueous solution of hydroiodic acid as deoxygenating reagent. The use of this procedure merits some additional comments. Alcohol deoxygenation processes are usually mediated by metals. The Barton–McCombie methodology is probably the most representative, although it involves several steps and the utilization of toxic tin hydride limits its application from an industrial point of view.^15^ Titanium(III) derivatives are gaining popularity in recent years as an alternative method, since they allow deoxygenation to be carried out in one step.^16^ Single-step metal-free deoxygenation, such as that employed here, is more challenging,^17^ due to the high stability of the C–O bond and kinetic inertia,^18^ and as a consequence it has been used less. In this context, it should be pointed out that Bro̷nsted acids, as hydroiodic, are promising deoxygenating agents due to their versatility.^19^ Once **3** was formed, the C(sp^3^)–H hydrogen atom was subsequently replaced by a methyl group, in the third step, to prevent the formation of trityl-type radicals. The replacement was executed in tetrahydrofuran, at −78 °C, by proton extraction with phenyllithium and subsequent capture of the resulting anion with methyl iodide. The methyl counterpart of **3**, 2-(1-(3-chlorophenyl)-1-phenylethyl)pyridine (**4**), was also obtained as a brown oil in almost quantitative yield.

Steps four and five constitute the second stage. Having generated in the first stage a phenyl with two substituents in 1,3-positions, a 2-benzylpyridine linked by the methylene unit and a chlorine atom, we approached the formation of the 2-phenylpyridine-type compound taking advantage of the presence of the chloride substituent. The latter was replaced by a pinacolboryl group (Bpin) in the fourth step, to subsequently perform a Suzuki–Miyaura cross-coupling reaction with 2-bromo-6-fluoropyridine in the fifth one. The borylation of **4**, which afforded 2-(1-phenyl-1-(3-pinacolborylphenyl)ethyl)pyridine (**5**), was executed with pinB-Bpin in the presence of 4 equiv of potassium acetate, at 130 °C, using 5 mol% of complex Pd(OMs){κ^2^-*C,N*-(C_6_H_4_-NH_2_)}(XPhos) (XPhos-Pd-G3; OMs = methanesulfonate, XPhos = 2-(dicyclohexylphosphino)-2′,4′,6′-triisopropyl-1,1′-biphenyl) as catalyst precursor,^20^ and dimethylformamide as solvent. Buchwald’s XPhos-Pd-G3 complex had previously proved to be efficient for the direct borylation of a variety of aryl halides.^21^ Although a black solid was formed under these conditions, probably due to decomposition of the catalyst precursor to palladium(0), the Miyaura-borylation of the aryl halide^22^ took place in an efficient manner after 3 h. Thus, the borylated product **5** was isolated as a light-yellow oleaginous gum in 61% yield, with previous purification of the reaction crude by silica column chromatography. The Suzuki–Miyaura cross-coupling^23^ between **5** and 2-bromo-6-fluoropyridine was carried out in a dioxane/water mixture as solvent, at 95 °C, using 10 mol% of the palladium derivative Pd(PPh_3_)_4_ as catalyst precursor, and 3 equiv of potassium carbonate as base. Under these conditions, the coupling was complete within 3 h. Thus, after purification of the reaction crude by silica column chromatography, the 2-phenypyridine-type compound 2-fluoro-6-(3-(1-phenyl-1-(pyridine-2-yl)ethyl)phenyl)pyridine (**6**) was isolated in 86% yield as a pale yellow oil.

The presence of the fluorine substituent at the pyridyl group of the 2-phenylpyridine moiety of **6** facilitated the attachment of a second 2-benzylpyridine in the last stage, which consists of only one step, the sixth. The nucleophilic aromatic substitution of this substituent by the anion 1-phenyl-1-(pyridin-2-yl)ethan-1-ide, resulting from the deprotonation of the tertiary C(sp^3^) atom of 2-(1-phenylethyl)pyridine, led to the designed molecule 2-(1-phenyl-1-(pyridin-2-yl)ethyl)-6-(3-(1-phenyl-1-(pyridin-2-yl)ethyl)phenyl)pyridine (**H**_**3**_**L**, **7**), in agreement with that previously observed for 2-fluoro-6-phenyl-pyridine.^10c^ The reaction was carried out in tetrahydrofuran, and the desired compound was obtained as a white solid in 41% yield, about 17% with regard to **1**, after a laborious workup including purification by silica column chromatography. Proligand **7** was formed as the mixture of diastereoisomers shown in Chart 1, which were found to be indistinguishable by NMR spectroscopy.

### Coordination of H_3_L to Iridium

**Scheme 2:**
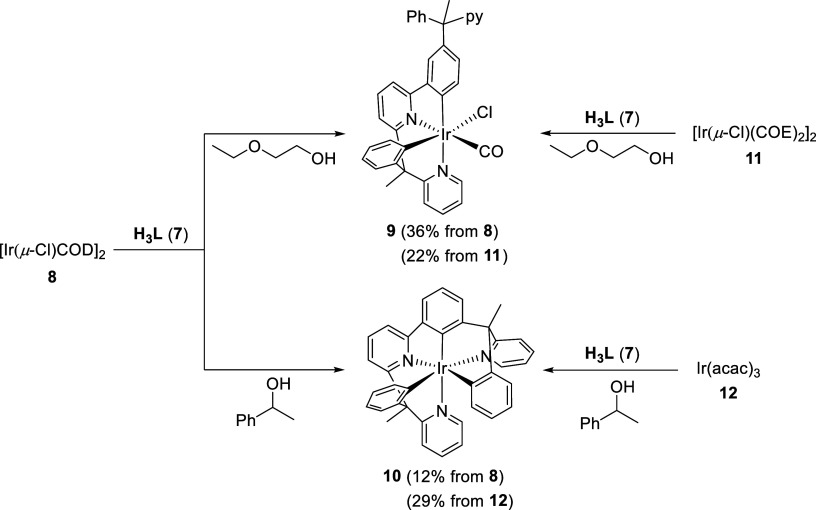
Synthesis of Complexes **9** and **10**

As a first option, we tested the well-known dimer [Ir(μ-Cl)(η^4^-COD)]_2_ (**8**, COD = 1,5-cyclooctadiene) as an iridium precursor. This compound had previously been shown to coordinate the heterocycles and promote the activation of an *ortho*-CH bond of the phenyl groups of the proligands 2-phenyl-6-(1-phenyl-1-(pyridin-2-yl)ethyl)pyridine^10c^ and 1-phenyl-3-(1-phenyl-1-(pyridin-2-yl)ethyl))isoquinoline.^10d^ In both cases, the products resulting from the assembly process presented the expected tetradentate 6-electron donor ligands (6tt′). However, the generated complexes depended on the reaction conditions and the primary or secondary character of the alcohol used as solvent. Mononuclear carbonyl derivatives [Ir(6tt′)Cl(CO)] were obtained with the primary 2-ethoxyethanol, at reflux, while the secondary character of 1-phenylethanol prevented the decarbonylation of the alcohol^24^ and allowed the formation of dimers [Ir(μ-Cl)(6tt′)]_2_. The nature of the alcohol used as solvent in the reaction of **8** with the new organic proligand **7** has a marked influence not only on the type of resulting product but also on the class of ligand generated, since two different coordination modes arise (Scheme 2).

**Figure 1:**
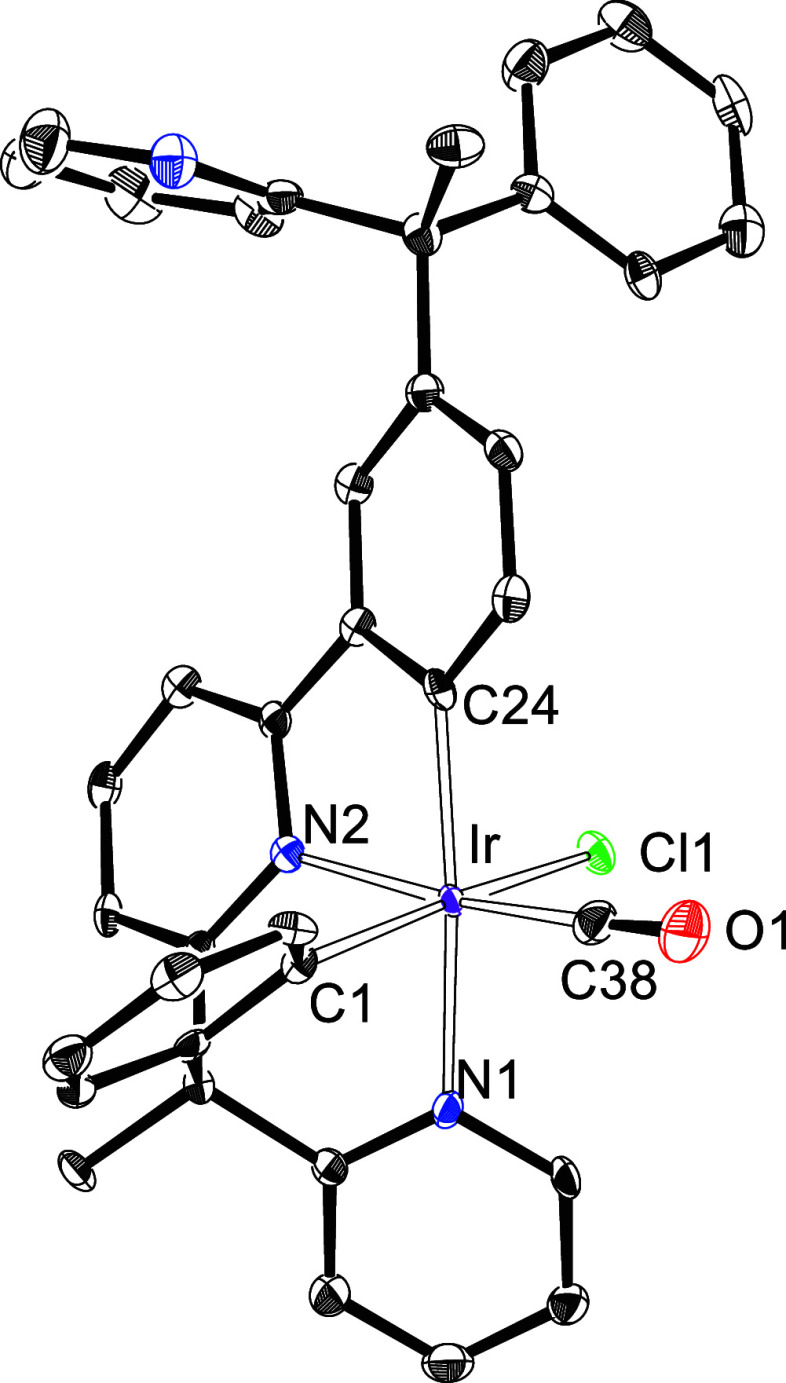
X-ray structure of **9** (50% probability ellipsoids, hydrogen atoms have been omitted). Selected bond lengths (Å) and angles (deg): Ir–C(1) = 2.029(5), Ir–C(24) = 2.033(5), Ir–N(1) = 2.137(4), Ir–N(2) = 2.058(4), Ir–Cl(1) = 2.4604(13), Ir–C(38) = 1.846(6); C(1)–Ir–Cl(1) = 167.87(15), C(38)–Ir–N(2) = 170.7(2), C(24)–Ir–N(1) = 168.42(18), N(1)–Ir–N(2) = 90.54(17), C(1)–Ir–C(24) = 98.7(2).

Treatment of suspensions of **8**, in 2-ethoxyethanol, with 2.0 equiv of **7**, under reflux, for 72 h afforded the carbonyl derivative Ir(κ^4^-*cis-C,C′-cis-N,N*′-HL)Cl(CO) (**9**), the [Ir(6tt′)Cl(CO)]-counterpart resulting from **7**. In a consistent manner with the previous [Ir(6tt′)Cl(CO)] complexes, its formation involves the selective orthometalation of the benzylpyridine moiety attached to the pyridyl group of the 2-phenylpyridine unit, in addition to the orthometalation of the latter and the alcohol decarbonylation. Complex **9** was separated as a yellow solid, in 36% yield, from the reaction crude, which contained a significant amount of decomposition products. The separation was performed by neutral alumina column chromatography. Figures S27 and S28 show the ^1^H and ^13^C{^1^H} NMR spectra of the solid, which reveal that the diastereoisomers resulting from the reaction **8** with both enantiomeric pairs **H**_**3**_**L**_**RR**_**-H**_**3**_**L**_**SS**_ and **H**_**3**_**L**_**RS**_**-H**_**3**_**L**_**SR**_ are also indistinguishable by NMR in this case. In addition, the complex was characterized by X-ray diffraction analysis. The structure (Figure 1) proves the preference of the iridium center by the benzylpyridine moiety attached to the pyridyl group. The coordination around the metal center is the expected octahedral with the heterocycles of the 6tt′ ligand mutually *cis*-disposed (N(1)–Ir–N(2) = 90.54(17)°). The phenyl group of the 2-phenylipyridine unit is situated *trans* to the pyridyl ring of the 2-benzylpyridine moiety (C(24)–Ir–N(1) = 168.42(18)°), whereas the phenyl group of the latter locates *trans* to the chloride anion (C(1)–Ir–Cl = 167.87(15)°. For its part, the carbonyl ligand lies *trans* to the heterocycle of the 2-phenylpyridine unit (C(38)–Ir–N(2) = 170.7(2)°). In accordance with the presence of a carbonyl ligand in **9**, its IR spectrum displays a characteristic ν(CO) band at 2017 cm^–1^ and the ^13^C{^1^H} NMR spectrum in dichloromethane-*d*_*2*_ contains a singlet at 172.3 ppm.

**Figure 2:**
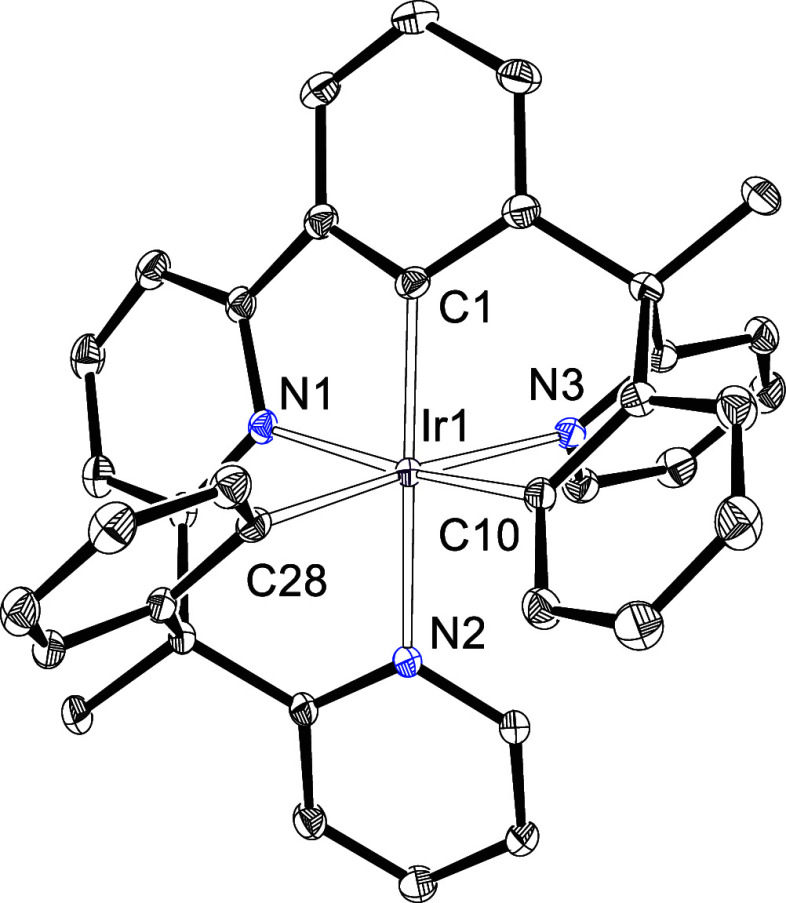
X-ray structure of the enantiomer Λ-Ir(κ^6^-*fac-C,C′,C″-fac-N,N*′*,N″-*L_RR_) of **10** (50% probability ellipsoids, hydrogen atoms have been omitted). Selected bond lengths (Å) and angles (deg) for both enantiomers: Ir(1)–C(1) = 1.986(3), 1.981(2), Ir(1)–C(10) = 2.030(3), 2.018(3), Ir(1)–C(28) = 2.035(3), 2.028(3), Ir(1)–N(1) = 2.087(2), 2.094(2), Ir(1)–N(2) = 2.099(2), 2.097(2), Ir(1)–N(3) = 2.148(2), 2.147(2); N(1)–Ir(1)–C(10) = 167.82(9), 166.74(9), N(2)–Ir(1)–C(1) = 166.72(10), 166.18(10), N(3)–Ir(1)–C(28) = 175.39(9), 173.73(9).

The use of 1-phenylethanol instead of 2-ethoxyethanol allowed the orthometalation of both benzylpyridine substituents of the 2-phenylpyridine core of **7**. Given the presence of two chiral centers in each isomer of the racemic mixture, enantiomeric pairs of four different diasteroisomers are possible in the resulting complex [Ir(9h)] (9h = 9-electron donor hexadentate ligand), one of them with a *fac* disposition of carbons and heteroatoms and the others displaying a *mer* arrangement (Chart S1). The former should be a consequence of the selective reaction of **H**_**3**_**L**_**RR**_**-H**_**3**_**L**_**SS**_ with **8**, while the other three would result from reactions of both **H**_**3**_**L**_**RR**_**-H**_**3**_**L**_**SS**_ and **H**_**3**_**L**_**RS**_**-H**_**3**_**L**_**SR**_. DFT calculations (B3LYPG-D3//SDD(f)6–31G**) revealed that the *fac* isomer is the most stable. In agreement with this, treatment of suspensions of **8**, in 1-phenylethanol, with 2.0 equiv of **7**, under reflux, for 72 h led to a brown solid, from which the calculated *fac* isomer Ir(κ^6^-*fac-C,C′,C″-fac-N,N*′*,N″-*L) (**10**) was separated as an orange solid, in 12% yield, by neutral alumina column chromatography. Its formation was supported by the ^1^H and ^13^C{^1^H} NMR spectra of the obtained solid (Figures S29–S30) and X-ray diffraction analysis. The structure, which contains both crystallographically independent enantiomers (Λ-Ir(κ^6^-*fac-C,C′,C″-fac-N,N*′*,N″-*L_RR_) and Δ-Ir(κ^6^-*fac-C,C′,C″-fac-N,N*′*,N″-*L_SS_)) in the asymmetric unit, demonstrates the coordination of the three heterocycles to the iridium center along with the orthometalacion of the three phenyl groups, as well as the *fac* dispositions of both types of atoms, carbon and nitrogen. Figure 2 gives a view of the enantiomer Λ-Ir(κ^6^-*fac-C,C′,C″-fac-N,N*′*,N″-*L_RR_). The angles N–Ir–C, which lie in the range 166–175°, are consistent with a high molecular stability.

Having established that the proligand **7** is able to generate complexes bearing 6tt′ and 9h ligands, depending upon the reaction solvent, we decided to address the improvement of the yields for the preparation of the complexes stabilized by such ligands. We had observed that the use of the cyclooctene (COE) derivative [Ir(μ-Cl)(η^2^-COE)]_2_ (**11**) instead of **8** facilitates the coordination of the proligand 1-phenyl-3-(1-phenyl-1-(pyridin-2-yl)ethyl))isoquinoline to the iridium center, increasing the yield for the preparation of the corresponding dimer [Ir(μ-Cl)(6tt′)]_2_ from 34% to 84%.^10d^ Such surprising improvement prompted us to change the precursor for the reactions shown in Scheme 2. Unfortunately, none of them improved. Using precursor **11** as starting material, the yield in the formation of **9** decreased to 22%, whereas complex **10** was not formed. An alternative precursor sometimes successfully used to synthesize iridium(III) compounds bearing a hexadentate ligand is the tris(acetylacetonate) derivative Ir(acac)_3_ (**12**).^12a,12c,12e^ Its use doubled the yield of the preparation of **10**, which reached 29% (Scheme 2). The reason for the relatively low yields obtained in the formation of **10**, with the investigated precursors, appears to be related to the fact that only half of **7**, **H**_**3**_**L**_**RR**_**-H**_**3**_**L**_**SS**_, is capable of forming the product.

### Photophysical and Electrochemical Properties of **9** and **10**

**Figure 3:**
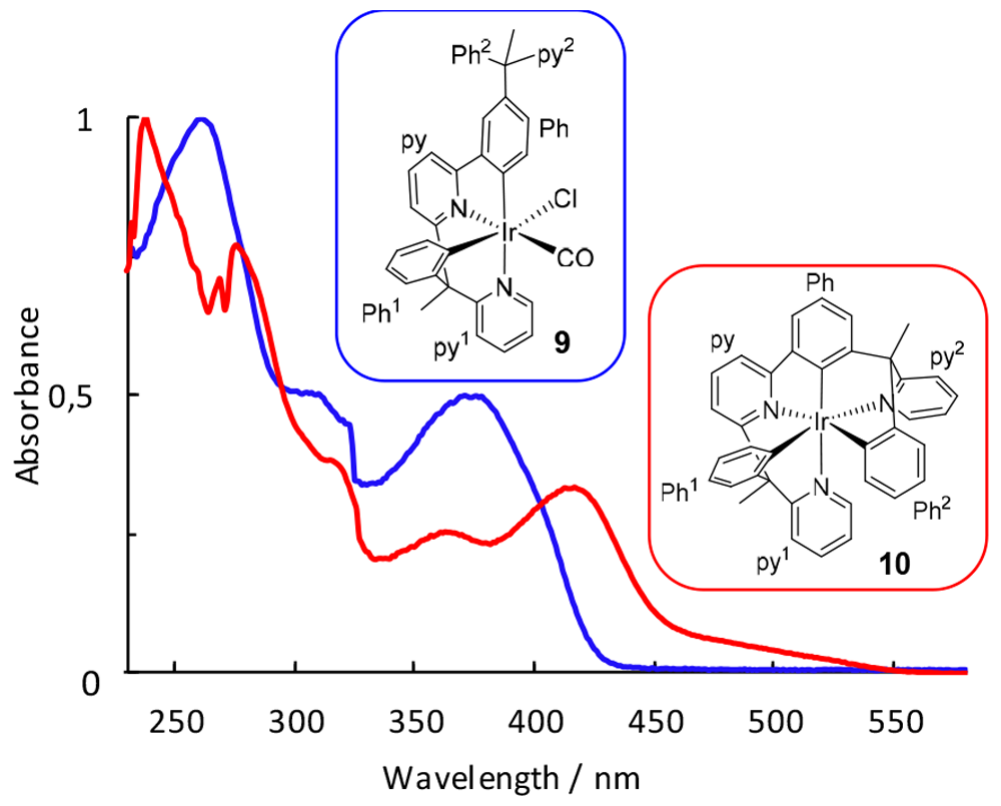
UV–vis absorption spectra of complexes **9** and **10** recorded in a 2-MeTHF solution (1.0 × 10^–4^ M) at 298 K.

The ultraviolet–visible (UV–vis) spectra of 2-methyltetrahydrofuran (2-MeTHF) solutions of **9** and **10** are typical for six-coordinate iridium(III) species (Figure 3), showing the usual three energy regions: <300, 350–450, and >450 nm. Table 1 points out some characteristic transitions, which were assigned based on DFT (TD-DFT) calculations (B3LYP-D3//SDD(f)/6–31G**) in THF (Figures S1 and S2, Tables S1 and S2). Higher energy bands (<300 nm) are due to ^1^π–π* transitions in the ligand, which take place without metal participation. Spin allowed charge transfers from the iridium center to the heterocycles combined with transitions from the phenyl groups to the heterocycles are observed between 350 and 450 nm, whereas formally spin forbidden transitions are also evident after 450 nm. They result from the large spin–orbit coupling produced by the iridium presence and mainly occur from the HOMO to the LUMO. The HOMO of both compounds delocalizes between the metal center and the phenyl groups, while the LUMO covers the heterocycles (Figures S5 and S6, Tables S3 and S4).

**Table 1:** Selected UV–vis Absortions for **9** and **10** (in 2-MeTHF) and Computed TD-DFT Vertical Excitation Energies (in THF)

λ_exp_ (nm)	ε (M^–1^ cm^–1^)	excitation energy (nm)	oscillator strength, *f*	transition	assignment
Complex **9**					
261	15 160	267	0.0312	HOMO-1 → LUMO+3 (39%), HOMO-9 → LUMO (11%)	Ph + Ph^1^ + Ph^2^ + py^2^ + Cl → py + py^1^
305	7660	299	0.0348	HOMO-1 → LUMO+1 (76%)	Ir + Ph + Ph^1^ → py^1^
376	7560	391 (S_1_)	0.0262	HOMO → LUMO (97%)	Ir + Ph + Ph^1^ → py + py^1^
455	100	452 (T_1_)	0	HOMO → LUMO (61%), HOMO-1 → LUMO (12%)	Ir + Ph + Ph^1^ → py + py^1^
Complex **10**					
274	16 915	267	0.0061	HOMO-7 → LUMO+1 (72%)	Ph^1^ + Ph^2^ → py^1^ + py^2^
361	557	370	0.0473	HOMO-1 → LUMO+2 (73%)	Ir + Ph^2^ → py + py^2^
419	7330	411	0.0819	HOMO-1 → LUMO (86%)	Ir + Ph^2^ → py + py^1^
455	1870	457 (S_1_)	0.0565	HOMO → LUMO (97%)	Ir + Ph → py + py^1^
509	70	499 (T_1_)	0	HOMO → LUMO (83%)	Ir + Ph → py + py^1^

DFT-calculated HOMO energy levels agree with those experimentally obtained from the electrochemical study of both complexes (Table 2). Figure S4 depicts the voltammograms, which were measured in acetonitrile, under argon, using [Bu_4_N]PF_6_ as supporting electrolyte (0.1 M). They display an Ir(III) to Ir(IV) oxidation, which appears at 1.10 V for **9** and at 0.14 V for **10**, versus Fc/Fc^+^. The high anodic potential observed for **9** agrees well with those reported for the [Ir(6tt′)Cl(CO)] complexes previously characterized (≈ 1.1 V),^10c,10d^ whereas the anodic potential of **10** compares well with those reported for emitters of classes [3b+3b′+3b″] and [6tt′+3b].^4,10d^ The notable difference between both values can be associated with the presence of the carbonyl ligand in **9**, which significantly stabilizes the HOMO. The oxidation of **9** is irreversible, while that of **10** is quasi-reversible. The irreversible character of the oxidation of **9** is not surprising. In this context, it should be noted that the carbonyl ligand is acidic and therefore should destabilize the oxidized species. Reductions were not detected within the acetonitrile window, between −2.9 and 1.6 V.

**Table 2:** Electrochemical and DFT MO Energy Data for Complexes **9** and **10**

		obs (eV)	calcd (eV)		
complex	*E*^ox^^a^ (V)	HOMO^b^	HOMO^c^	LUMO^c^	HLG^c^
**9**	1.10^d^	–5.91	–5.64	–1.74	3.90
**10**	0.14^e^	–4.94	–4.75	–1.29	3.46

a Measured under argon in acetonitrile/[Bu_4_N]PF_6_ (0.1 M), vs Fc/Fc^+^.

b HOMO = – [E^ox^ vs Fc/Fc^+^ + 4.8] eV.

c Values from DFT calculations.

d Anodic potential (irreversible oxidation).

e *E*_1/2_^ox^ (quasi-reversible oxidation).

**Figure 4:**
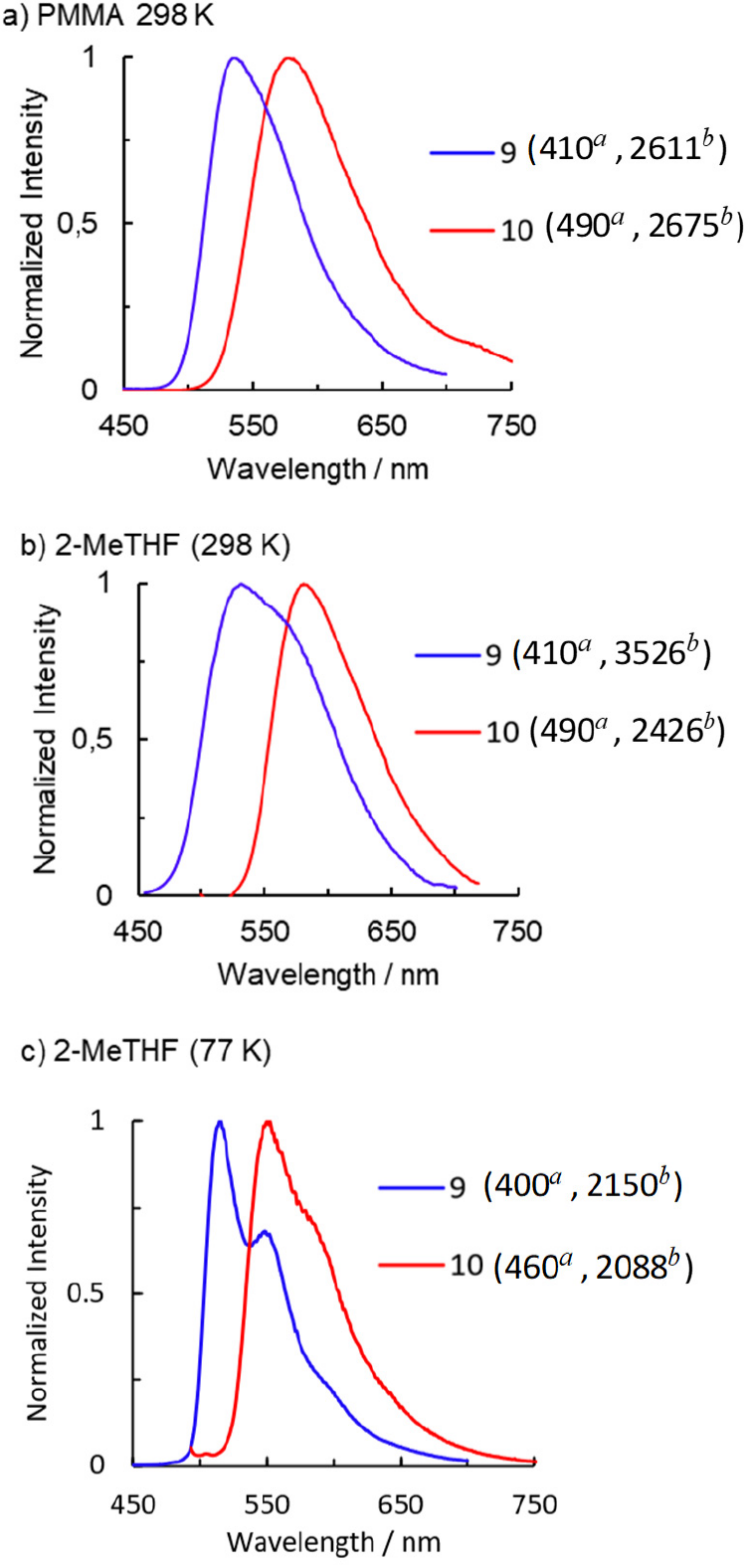
Emission spectra of **9** and **10** in (a) 5 wt% PMMA films at 298 K, (b) 2-MeTHF at 298 K, and (c) 2-MeTHF at 77 K. ^*a*^λ_exc_/nm; ^*b*^fwhm/cm^–1^.

Complexes **9** and **10** are phosphorescent emitters upon photoexcitation. The measurements were performed in a doped poly(methyl methacrylate) (PMMA) film at 5 wt%, at room temperature, and in 2-MeTHF at room temperature and at 77 K. Figure 4 collects the spectra recorded under such conditions. Emissions occur from the respective T_1_ excited states, as is suggested by excellent agreement between the experimental wavelengths and the values calculated in THF for the difference in energy between the optimized triplet states T_1_ and the singlet states S_0_.

The shape of the emission bands is broadly similar in both compounds. Full width at half-maximum (fwhm) values depend on the medium and lie in the range 2150–3526 cm^–1^ for **9** and 2088–2675 cm^–1^ for **10**. The main difference between the spectra is observed in the energy of the emission maxima. Complex **9** is a green emitter with maxima between 515 and 549 nm. The presence of the carbonyl group in the latter produces an increase of the HOMO–LUMO gap with respect to **10** (Table 2). Thus, complex **10** with a smaller gap between frontier orbitals emits in the lower energy yellow region of the spectrum, with maxima between 552 and 587 nm. The lifetimes are short, lying in the range 0.6–8.4 μs. Quantum yields are moderate, about 0.40, and similar for both compounds in PMMA. While for **10** the value is maintained in 2-MeTHF at room temperature, for **9** it is reduced by half. Such a decrease is associated with a decrease in the radiative rate constant (*k*_r_) for **9** of about 1 order of magnitude with respect to the value observed for **10**. This suggests significant differences in the solvation of both compounds, which tentatively could be related to the different nature of the polydentate ligands. As a consequence of this situation, the ratio between the radiative and nonradiative (*k*_*nr*_) rate constants is the same for both compounds in PMMA and also in solution for **10** (0.7). In contrast to **10**, the ratio decreases to 0.2 for **9** in 2-MeTHF (Table 3).

**Table 3:** Emission Data for **9** and **10**

Complex	calcd λ_em_ (nm)	Media (*T*/K)	λ_em_ (nm)	τ (μs)	Φ	*k*_r_^a^ (s^–1^)	*k*_nr_^a^ (s^–1^)	*k*_r_/*k*_nr_
**9**	507	PMMA (298)	529	1.7	0.43	2.5 × 10^5^	3.4 × 10^5^	0.7
		2-MeTHF (298)	535	3.7	0.18	4.9 × 10^4^	2.2 × 10^5^	0.2
		2-MeTHF (77)	515, 549	8.4				
**10**	566	PMMA (298)	576	1.4	0.41	2.9 × 10^5^	4.2 × 10^5^	0.7
		2-MeTHF (298)	581	0.6	0.42	7.0 × 10^5^	9.5 × 10^5^	0.7
		2-MeTHF (77)	552, 587	7.7				

a Calculated according to *k*_r_ = ϕ/τ and *k*_nr_ = (1 – ϕ)/τ.

### Electroluminescence of OLED Devices Based on **10**

**Figure 5:**
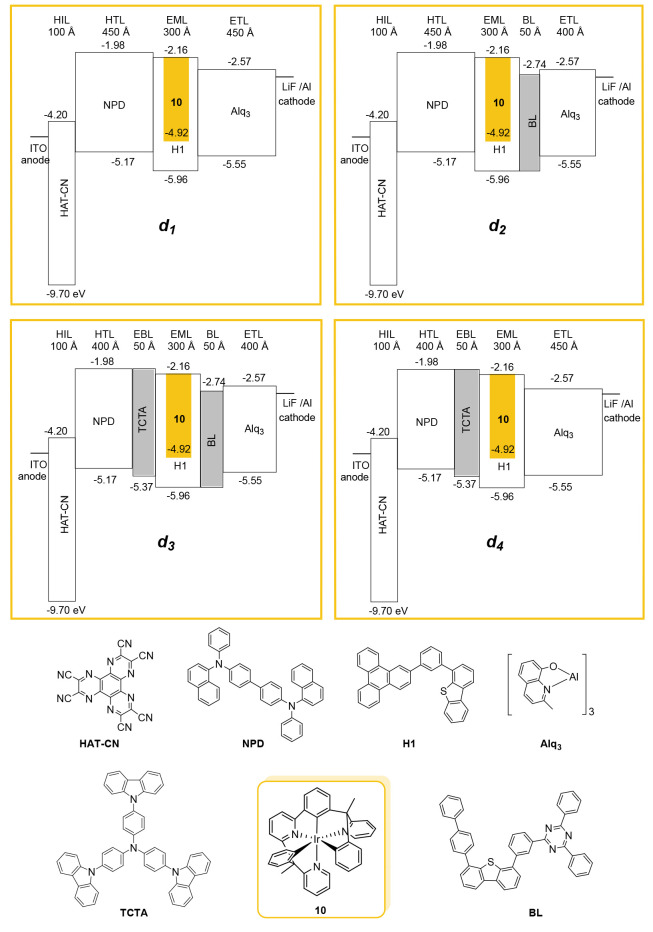
Energy levels and materials used in the different layers of the devices.

To investigate the applicability of encapsulated-type *pseudo*-tris(heteroleptic) iridium(III) compounds in OLED device fabrication, we studied the behavior of complex **10** in four bottom-emission OLED structures, as an example of a yellow phosphorescent emitter. Figure 5 outlines the configurations of the devices ***d*_*1*_***–****d***_***4***_, including the energy levels and the thickness of the layers, as well as the chemical nature of the materials used.

The devices were built by high vacuum (<10^–7^ Torr) thermal evaporation. Immediately after their fabrication, they were encapsulated within a nitrogen glovebox (<1 ppm of H_2_O and O_2_) and a moisture getter was incorporated inside the package, which was closed with a glass lid that was subsequently sealed with an epoxy resin. The anode and cathode electrodes of the four devices were set up with the same components. The anode consisted of 750 Å of indium tin oxide (ITO), while the cathode was formed by a 10 Å LiF electron injection layer followed by another 1000 Å Al layer. The simplest device structure (***d*_*1*_**) consisted of the following layers sequentially disposed from the ITO surface to the cathode: 100 Å of HAT-CN as the hole injection layer (HIL), 450 Å of NPD as a hole transporting layer (HTL), 300 Å of an emissive layer (EML) containing host (H1) doped with complex **10** as an emitter at 9%, and 450 Å of Alq_3_ as an electron transporting layer (ETL). In the search for improvement to the ***d*_*1*_** features, we also fabricated devices ***d*_*2*_***–****d*_*4*_**, resulting from modifications of the ***d*_*1*_** structure (Figure 5). The ***d*_*2*_** device was made with the aim of preventing holes and excitons from leaking into the low triplet Alq_3_ electron transport layer, quenching them. With this goal, a 50 Å layer of the hole-blocking material BL (T_1_ = 2.56 eV) was assembled between the emissive and Alq_3_ layers. At the same time, the thickness of the latter was reduced to 400 Å. The ***d*_*3*_** device displays the most complete structure. It incorporates, between the hole transporting and emissive layers of ***d*_*2*_**, a 50 Å electron and excitons blocking layer of the high triplet energy TCTA compound (T_1_ = 2.76 eV), simultaneously reducing the thickness of the hole transporting layer to 400 Å. This additional layer (EBL) should prevent exciton leakage to the low triplet NPD compound, which would improve the device EQE. To assess the relative relevance of both introduced layers, we finally built ***d*_*4*_** removing the BL layer from ***d*_*3*_** and increasing the thickness of the electron transporting layer to 450 Å. The total thickness of the organic stack remained constant across all four devices. Such design was carried out to eliminate possible distortions in the efficiency measurement due to changes in the outcoupling related to the thickness of the organic layers. Table 4 summarizes the performance of the devices, including emission features and values of turn-on voltage, luminous efficacy (LE), external quantum efficiency (EQE), and power efficacy (PE) at a luminance of 600 cd m^–2^

**Table 4:** EL Performance of the Devices

	1931 CIE				at 600 cd m^–2^			
device	*x*	*y*	λ_max_ (nm)	fwhm (nm)	voltage (V)	LE (cd A^–1^)	EQE (%)	PE (lm W^1–^)
***d*_*1*_**	0.520	0.477	576	84	6.6	21.4	7.8	10.2
***d*_*2*_**	0.519	0.478	576	84	6.7	30.3	11	14.1
***d*_*3*_**	0.517	0.479	576	84	7.1	31.3	11.3	13.8
***d*_*4*_**	0.518	0.478	576	85	6.9	23.2	8.4	10.6

**Figure 6:**
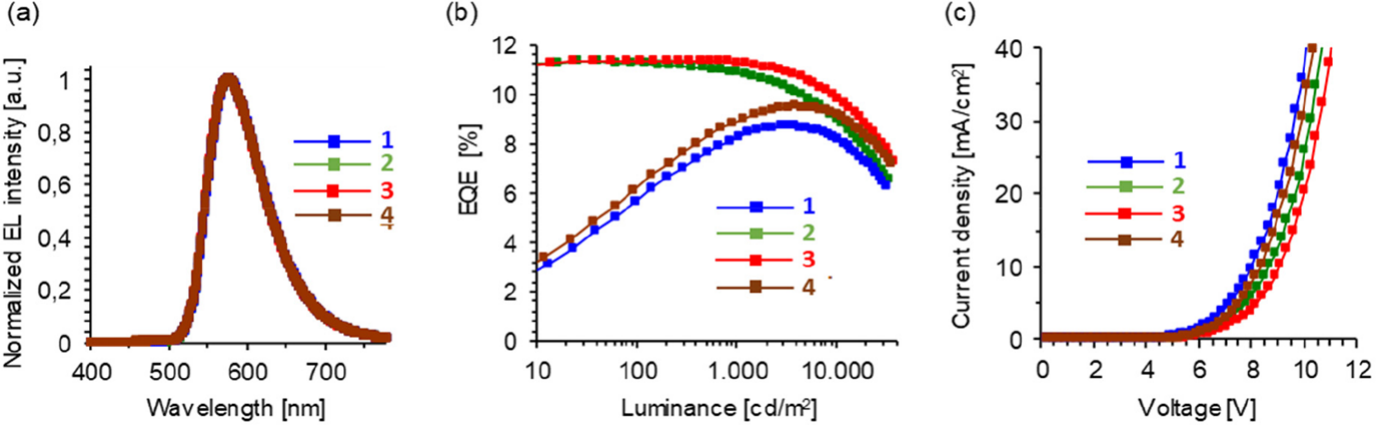
(a) Electroluminescence (EL) spectra of the devices measured at 10 mA/cm^2^. (b) EQE vs luminance. (c) Current density vs voltage.

Complex **10** provided a yellow emission with 1931 CIE (x:y) ∼ (0.52:0.48), wavelength maximum at 576 nm, full width half-maximum of 84 nm, and emission offset below 525 nm (Figure 6a). The finding corresponds to a triplet emission energy of the emitter of more than 2.35 eV. To efficiently confine these high triplet excitons, layers of high triplet material are required around the emissive one. However, both the NPD compound that acts as a hole transporting (T_1_ = 2.29 eV) and the Alq_3_ derivative that proceeds as an electron carrier (T_1_ ∼2.0–2.1 eV) have a lower triplet, which cannot efficiently confine the excitons within the emissive layer. As a consequence, the simplest device structure ***d*_*1*_**, without the protecting layers of BL and TCTA compounds, has the lowest EQE of 7.8%. As expected, the introduction of the BL hole blocking material between the emissive and Alq_3_ layers prevents excitons leaking to the latter. As a consequence, the ***d*_*2*_** configuration improves the device EQE by about 40% with regard to ***d*_*1*_**, reaching a value of 11.0%. A further slight improvement is achieved with the exciton and electron blocking layer of TCTA, between the emissive and hole transporting layers. Thus, the ***d*_*3*_** device shows an EQE of 11.3%, the highest value of the four. The BL effect is significantly greater than the TCTA effect in EQE improving, as demonstrated by the ***d*_*4*_** device. The latter, bearing the electron and exciton blocking TCTA compound as a unique additional layer, achieves an EQE of 8.4%, a significantly poorer value than that of the device ***d*_*2*_** containing the BL hole blocking layer. This could be explained by the location of the recombination zone in the emissive layer. As follows from the energy levels shown in Figure 5, holes are being transported via emissive layer by the emitter, while the host transports electrons. Thus, the most likely recombination zone is in the proximity to the ELT side of the emissive layer rather than to HTL side. Figure 6b gives additional evidence of the marked difference in effect between the layers of BL and TCTA compounds on the EQE of devices, as a consequence of the localization of the recombination region of the emissive layer close to the ETL side. At low luminance (10–100 cd m^–2^) the efficiency of devices ***d*_*1*_** and ***d*_*4*_**, which do not bear BL layer, is very low and significantly increases with luminance increase, whereas the efficiency of devices ***d*_*2*_** and ***d*_*3*_**, containing BL layer, remains very high even at low luminance. However, the recombination zone appears to propagate toward the HTL side of the emissive layer with current density and luminance increase. As a result, the effect of BL and TCTA compounds on the device efficiency is approximately the same, at luminance level above 10000 cd m^–2^. The four devices display very similar profiles for current density versus voltage showing turn-on voltages between 6.6 and 7.1 V (Figure 6c).

## Concluding Remarks

This study reveals that encapsulated-type *pseudo*-tris(heteroleptic) iridium(III) emitters of class [Ir(9h)], with a *fac*-disposition of carbon and nitrogen atoms, are accessible when the organic molecule responsible of the formation of the hexadentate ligand h is properly designed and can be prepared. Once the organic proligand is isolated, several details should be taken into account for its coordination to the iridium center, the process that gives rise to the synthesis of the emitter. Such details are related to the metal precursor and the reaction solvent. Although the well-known dimer [Ir(μ-Cl)(η^4^-COD)]_2_ is suitable as an iridium precursor, the tris(acetylacetonate) derivative Ir(acac)_3_ is a more appropriate starting material. Secondary alcohols of high boiling point are preferred solvents over primary alcohols, since the former prevent a possible metal carbonylation that could inhibit the full coordination of the proligand. As here demonstrated, these emitters have applicability in the fabrication of OLED devices, as the device performance is more than reasonable.

In summary, we here report the overall process up to the fabrication of a yellow emitting device, which bears the first encapsulated-type *pseud*o-trisheteroleptic iridium(III) emitter and displays a maximum wavelength of 576 nm, an external quantum efficiency of 11.3%, and a luminous efficacy of 31.3 cd A^–1^ at 600 cd m^–2^.

## Experimental Section

[Ir(μ-Cl)(η^4^-COD)]_2_ (**8**)^27^ and [Ir(μ-Cl)(η^2^-COE)_2_]_2_ (**11**)^28^ were prepared by published methods. Ir(acac)_3_ was purchased from Strem Chemicals. Commercially available reagents were used without further purification. Chemical shifts and coupling constants in the NMR spectra (Figures S14–S30) are given in ppm and Hz, respectively.

### Synthesis of (3-Chlorophenyl)(phenyl)(pyridine-2-yl)methanol (**2**)

A solution of 1-bromo-3-chlorobenzene (2.50 g, 12.38 mmol) in 80 mL of THF was cooled to −78 °C, and *n-*BuLi (1.6 M solution in hexane, 8.64 mL, 13.62 mmol) was added dropwise. The anion was stirred for 1 h to perform the lithium halogen exchange. A solution of 2-benzoylpyridine (1.45 mL, 12.38 mmol) in THF was added dropwise, keeping the temperature below −60 °C. The reaction was stirred over 1 h at −78 °C and then allowed to warm to room temperature overnight. The reaction flask was immersed in an ice bath, water (ca. 80 mL) was added to the reaction, and the pH was adjusted to 5 with citric acid. The aqueous phase was extracted with EtOAc (3 × 20 mL), and the combined organic extracts were washed with brine and dried over MgSO_4_, before filtering and evaporation to afford **2** (3.87 g, quantitative yield) as a dark orange/brown oil. Anal. Calcd for C_18_H_14_ClNO: C, 73.10; H, 4.77; N, 4.74. Found: C, 72.80; H, 5.07; N, 4.57. HRMS (electrospray, *m*/*z*): calcd for C_18_H_14_ClNNaO [M + Na]^+^ 318.0656; found, 318.0654. ^1^H NMR (300 MHz, DMSO-*d*_*6*_): δ 8.56 – 8.54 (m, 1H), 7.83 – 7.78 (m, 1H), 7.74 – 7.70 (m, 1H), 7.41 (m, 1H), 7.36 – 7.22 (m, 9H), 6.80 (s, 1H, OH). ^13^C{^1^H}-NMR (75 MHz, DMSO-*d*_*6*_): δ 164.4, 149.6 (both C_q_), 148.0 (CH), 146.6 (C_q_), 136.7 (CH), 132.3 (C_q_), 129.3 (CH), 127.8 (2CH), 127.6 (3CH), 126.9, 126.8, 126.6, 122.1, 121.4 (all CH), 80.4 (COH).

### Synthesis of 2-((3-Chlorophenyl)(phenyl)methyl)pyridine (**3**)

Hydroiodic acid (57% aq., d = 1.70 g/mL, 7.9 mL, 60 mmol) was added to a solution of **2** (1.7 g, 5.75 mmol) in 24 mL of acetic acid, and the mixture was heated to 100 °C for 3 h. The reaction was concentrated in vacuo to a thick slurry, water/ice was added, and the solution was made to pH 8–9 with solid sodium carbonate. The product was extracted into EtOAc (3 × 20 mL), and the organic phases were combined, washed with water and brine, and dried over MgSO_4_. The organic extracts were filtered through a thin pad of silica/celite and concentrated in vacuo to a brown oil. The crude was taken into dichloromethane and adsorbed onto silica and then purified by column chromatography with silica gel eluting with 5–40% EtOAc/pentane to afford **3** as a brown oil (1.02 g, 83%). Anal. Calcd for C_18_H_14_ClN: C, 77.28; H, 5.04; N, 5.01. Found: C, 77.57; H, 5.01; N, 4.94. HRMS (electrospray, *m*/*z*): calcd for C_18_H_15_NCl [M + H]^+^ 280.0888; found, 280.0878. ^1^H NMR (300 MHz, DMSO-*d*_*6*_) δ 8.59–8.57 (m, 1H), 7.83–7.78 (m, 1H), 7.38–7.15 (m, 12H), 5.75 (s, 1H). ^13^C{^1^H}-NMR (75 MHz, DMSO-*d*_*6*_): δ 161.2 (C_q_), 148.9 (CH), 145.3, 142.2 (both C_q_), 137.6 (CH), 132.9 (C_q_), 130.2, 129.0 (2C), 128.8, 128.5 (2C), 127.9, 126.6, 126.4, 124.0, 122.1 (all CH), 56.9 (CH Csp^3^).

### Synthesis of 2-(1-(3-Chlorophenyl)1-phenylethyl)pyridine (**4**)

A solution of **3** (1.02 g, 3.65 mmol) in 8 mL of THF was cooled to −78 °C and a solution of PhLi (1.9 M in dibutyl ether, 3.06 mL, 5.85 mmol) was added dropwise, maintaining the temperature below −50 °C. The anion was stirred for 45 min before methyl iodide (364 μL, 5.85 mmol) was added via syringe, again maintaining the temperature below −50 °C. The reaction was stirred at −78 °C for 30 min and then allowed to warm slowly and stirred overnight at rt. Water was added until the reaction became turbid, and the product was partitioned into EtOAc. The layers were separated, and the aqueous phase was extracted with EtOAc (3 × 20 mL) before the organic extracts were combined and washed with brine followed by drying over MgSO_4_. The organic extracts were filtered through a thin pad of celite and concentrated in vacuo to give **4** as a brown oil (1.07 g, quantitative yield). Anal. Calcd for C_19_H_16_ClN: C, 77.68; H, 5.49; N, 4.77. Found: C, 77.46; H, 5.79; N, 4.33. HRMS (electrospray, *m*/*z*): calcd for C_19_H_17_ClN [M + H]^+^ 294.1044; found, 294.1039. ^1^H NMR (300 MHz, DMSO-*d*_*6*_): δ 8.59–8.56 (m, 1H), 7.72–7.61 (m, 1H), 7.32–7.24 (m, 6H), 7.07–7.00 (m, 5H), 2.15 (s, 3H, Me). ^13^C{^1^H}-NMR (75 MHz, DMSO-*d*_*6*_): δ 166.1, 154.4 (both C_q_), 148.4 (CH), 147.8, 138.7 (both C_q_), 137.2, 136.0, 128.9, 128.6 (2C), 127.9 (2C), 126.0, 123.3, 122.0, 121.3, 117.5 (all CH), 57.5 (*C*Me), 28.0 (Me).

### Synthesis of 2-(1-Phenyl-1-(3-pinacolborylphenyl)ethyl)pyridine (**5**)

A mixture of **4** (0.91 g, 3.1 mmol), bis(pinacolato)diboron (1.02 g, 4.03 mmol), potassium acetate (1.22 g, 12.4 mmol), and (2-dicyclohexylphosphino-2′,4′,6′-triisopropyl-1,1′-biphenyl)[2-(2′-amino-1,1′-biphenyl)]palladium(II) methanesulfonate (Pd-G3-X-Phos, 131 mg, 0.15 mmol), in 15 mL of DMF, was heated to 130 °C for 3 h. During this time, the reaction turned dark brown and homogeneous before a black precipitated appeared (presumably Pd catalyst). The reaction was concentrated to 50% volume and then poured over ice/water and the product extracted into EtOAc (4 × 15 mL). The layers were separated, and the organic phase was filtered through celite and then washed with warm water and brine to remove any DMF traces. The organic phase was dried over MgSO_4_, filtered, and concentrated in vacuo. The crude was purified by column chromatography with silica gel starting with 100% pentane and increasing the concentration of AcOEt to 15% to give a light-yellow oleaginous gum (813 mg, 61%). Anal. Calcd for C_25_H_28_BNO_2_: C, 77.93; H, 7.32; N, 3.64. Found: C, 77.79; H, 7.78; N, 3.26. HRMS (electrospray, *m*/*z*): calcd for C_25_H_28_BNNaO_2_ [M + Na]^+^ 408.2110; found, 408.2104. ^1^H NMR (300 MHz, DMSO-*d*_*6*_): δ 8.57–8.54 (m, 1H), 7.72–7.67 (m, 1H), 7.55–7.52 (m, 1H), 7.40–7.37 (m, 1H), 7.30–7.23 (m, 5H), 7.16–7.12 (m, 2H), 7.05–7.01 (m, 2H), 2.13 (s, 3H, C*Me*), 1.25 (br s, 12H, Me Bpin). ^13^C{^1^H}-NMR (75 MHz, CD_2_Cl_2_): δ 149.2 (CH), 149.0, 148.1, 147.0, 146.2 (all C_q_), 136.2, 134.6, 132.9, 132.4, 131.5, 129.1, 128.3, 127.9, 127.7, 126.4, 123.7, 121.5 (all CH), 84.2 (C_q_, Bpin), 55.5 (*C*_q_Me), 29.7 (C_q_*Me*), 25.1 (Me Bpin).

### Synthesis of 2-Fluoro-6-(3-(1-phenyl-1-(pyridine-2-yl)ethyl)phenyl)pyridine (**6**)

A mixture of **5** (800 mg, 2.08 mmol), 2-bromo-6-fluoropyridine (320 μL, 3.12 mmol), Pd(PPh_3_)_4_ (360.5 mg, 0.312 mmol), and potassium carbonate (861 mg, 6.24 mmol) in dioxane/water (50 mL, 4:1) was heated at 95 °C for 3 h. The reaction was cooled at room temperature and diluted with water (ca. 30 mL). Then, the product was extracted into EtOAc (3 × 20 mL), and the organics extracts were combined and dried over MgSO_4_ before filtering and concentrating in vacuo to give a yellow oil. The crude material was loaded onto silica and purified by column chromatography, eluting with EtOAc/pentane increasing the polarity from 5% to 30% of EtOAc, to afford a pale-yellow oil (637 mg, 86%). Anal. Calcd for C_24_H_19_FN_2_: C, 81.33; H, 5.40; N, 5.36. Found: C, 81.56; H, 5.35; N, 5.05. HRMS (electrospray, *m*/*z*): calcd for C_24_H_19_FN_2_Na [M + Na]^+^ 377.1424; found, 377.1489. ^1^H NMR (300 MHz, DMSO-*d*_*6*_): δ 8.58 (ddd, *J* = 4.8, 2.0, 1.0 Hz, 1H), 8.07–7.96 (m, 1H), 7.80 (br s, 2H), 7.74–7.67 (m, 1H), 7.51–7.44 (m, 1H), 7.42–7.37 (m, 1H), 7.32–7.22 (m, 5H), 7.16–7.06 (m, 5H), 2.21 (s, 3H, Me). ^13^C{^1^H}-NMR (75 MHz, DMSO-*d*_*6*_): δ 166.1, 149.0 (both C_q_), 148.6 (CH), 148.0, 146.0, 139.7, 136.5 (all C_q_), 136.3, 130.6, 129.9, 128.5, 127.7, 127.6, 126.4, 125.6, 125.4, 124.5, 123.0, 121.4, 120.1, 118.4, 110.9 (all CH), 54.9 (*C*_q_Me), 29.2 (Me).

### Synthesis of 2-(1-Phenyl-1-(pyridin-2-yl)ethyl)-6-(3-(1-phenyl-1-(pyridin-2-yl)ethyl)phenyl)pyridine (H_3_L, **7**)

A suspension of lithium chloride (593 mg, 14.1 mmol) in THF was cooled to −78 °C, and LDA (1 M in THF, 2.82 mL, 2.82 mmol) was added. The reagents were stirred for 5 min, and then a solution of 2-(1-phenylethyl)pyridine (310 mg, 1.69 mmol) in THF (5 mL) was added. The anion was stirred for 3 h at −78 °C forming a red solution/suspension. At this temperature a solution of **6** (500 mg, 1.41 mmol) in THF (4 mL) was added forming a dark red/brown solution, which was stirred at −78 °C for 1 h and then allowed to warm to rt. During this time a red/burgundy solution forms, and the reaction was held at rt for 30 min and then heated to 70 °C overnight. The reaction was cooled to rt, and an additional amount of 2-(1-phenylethyl)pyridine (342 mg, 1.87 mmol) and lithium chloride (795.6 mg, 18.8 mmol) were added. The reaction mixture was then cooled back down to −78 °C. Then, more LDA (1 M in THF, 3.52 mmol, 3.52 mL) was added, and the reaction was stirred at −78 °C for 3 h before being allowed to warm to rt. The reaction was stirred at rt for 30 min and then heated to 70 °C for 2 days, forming a red/brown solution with a yellow precipitate. The reaction was cooled to rt and concentrated in vacuo to ca. 10% volume, and then water and EtOAc were added followed by heating to 60 °C to ensure full dissolution of the product. The biphasic mixture was filtered through celite to remove some flocculants solids. The layers were separated, and the aqueous phase was extracted with EtOAc. The organic extracts were combined and washed with water, and then brine, and finally dried over MgSO_4_ before filtering and concentrating in vacuo to provide a brown oil. It was loaded onto silica and purified by column chromatography, eluting with EtOAc/pentane increasing the polarity from 5% to 30% of EtOAc, to afford a white solid (298 mg, 41%). Anal. Calcd for C_37_H_31_N_3_: C, 85.85; H, 6.04; N, 8.12. Found: C, 85.56; H, 5.98; N, 8.17. HRMS (electrospray, *m*/*z*): calcd for C_37_H_32_N_3_ [M + H]^+^ 518.2591; found, 518.2598. ^1^H NMR (300 MHz, CD_2_Cl_2_): δ 8.60–8.57 (m, 1H), 8.55–8.52 (m, 1H), 7.80 (dt, *J* = 7.7, 1.4 Hz, 1H), 7.77–7.75 (m, 1H), 7.61 (t, *J* = 7.8 Hz, 1H), 7.55–7.48 (m, 3H), 7.33 (t, *J* = 7.8 Hz, 1H), 7.29–7.21 (m, 6H), 7.16–7.04 (m, 10H), 2.23, 2.22 (both s, 3H each, Me). ^13^C{^1^H}-NMR (75 MHz, CD_2_Cl_2_): δ 167.5, 167.3, 166.2, 155.9, 149.3 (all C_q_), 149.2, 148.9 (both CH), 148.7, 139.4 (both C_q_), 137.0, 136.2, 136.0, 129.7, 129.1 (4C), 128.5, 128.3 (4C), 127.9, 126.5, 126.4, 124.8, 124.1, 123.8, 123.8, 122.3, 121.5, 121.4, 117.8 (all CH), 58.3, 55.7 (both *C*_q_Me), 29.7, 28.4 (both Me).

### Preparation of IrCl(κ^4^-*cis*-*C,C′*-*cis*-*N,N′*-HL)(CO) (**9**)

***Route a:*** A mixture of **8** (80 mg, 0.118 mmol) and **H**_**3**_**L** (**7**) (244 mg, 0.473 mmol) in 5 mL of 2-ethoxyethanol was heated under reflux for 72 h, and a dark red solution was formed. Afterward, the solution was cooled down to rt and the solvent was removed under vacuum. The crude was washed several times with pentane (5 × 10 mL) to remove all the possible traces of 2-ethoxyethanol. The crude was purified by neutral alumina column chromatography using diethyl ether as eluent and finishing with a mixture of 3:1 diethyl ether/dichloromethane. A yellow solid was obtained. Yield: 62 mg (36%). ***Route b:*** The same procedure as that for route *a* was followed starting from a mixture of **11** (100 mg, 0.111 mmol) and **H**_**3**_**L** (**7**) (224 mg, 0.444 mmol). Yield: 38 mg (22%). Anal. Calcd for C_38_H_29_ClIrN_3_O: C, 59.17; H, 3.79; N, 5.45. Found: C, 59.18; H, 3.88; N, 5.66. HRMS (electrospray, *m*/*z*): calcd for C_38_H_29_IrN_3_O [M-Cl]^+^ 736.1936; found, 736.1911. IR (cm^–1^): ν(CO) 2017 (s). ^1^H NMR (300.15 MHz, CD_2_Cl_2_): δ 9.31 (d, *J* = 4.9 Hz, 1H), 8.60 (s, 1H), 7.99–7.88 (m, 3H), 7.79–7.69 (m, 2H), 7.59 (t, *J* = 7.5 Hz, 1H), 7.48 (d, *J* = 7.8 Hz, 1H), 7.46–7.38 (m, 2H), 7.37–7.35 (m, 1H), 7.28–7.21 (m, 3H), 7.23–7.21 (m, 1H), 7.17–7.14 (m, 4H), 7.00 (dt, *J* = 15.7, 8.1 Hz, 2H), 6.83 (t, *J* = 7.1 Hz, 1H), 2.61 (s, 3H), 2.24 (s, 3H). ^13^C{^1^H}-NMR (75 MHz, CD_2_Cl_2_): δ 172.3 (CO), 167.8, 167.2, 158.2 (2C) (all C_q_), 157.4, 149.4 (both CH), 149.1, 147.9, 144.7, 143.9 (all C_q_), 140.4, 139.7 (both CH), 138.7 (C_q_),136.6, 136.5 (both CH), 133.8 (C_q_), 132.0, 131.8, 129.3, 128.5, 127.4, 126.6, 125.5, 125.3, 125.0, 124.8, 124.7, 123.9, 123.8, 122.1, 121.6, 118.0, 117.8 (all CH), 59.8 (*C*_*q*_Me attached to Ph of ppy), 55.3 (*C*_*q*_Me attached to py of ppy), 29.8 (C_q_*Me* attached to Ph of ppy), 22.7 (C_q_*Me* attached to py of ppy).

### Preparation of Ir(κ^6^*-C,C′,C′′,N,N′,N′′-*L) (**10**)

***Route a:*** A mixture of **8** (75 mg, 0.111 mmol) and **H**_**3**_**L** (**7**) (224 mg, 0.444 mmol) in 5 mL of 1-phenyethanol was heated under reflux for 72 h, and a dark red solution was formed. Afterward, the solution was cooled to rt and the solvent was removed under vacuum. The crude was washed with diethyl ether (10 × 10 mL) to remove all the possible traces of 1-phenylethanol. The brown solid crude was purified by neutral alumina column chromatography using diethyl ether as eluent and finishing with a mixture of 3:1 diethyl ether/dichloromethane. An orange solid was obtained. Yield: 20 mg (12%). ***Route b:*** The same procedure as that for route *a* was followed starting from a mixture of **12** (93 mg, 0.189 mmol) and **H**_**3**_**L** (**7**) (196.3 mg, 0.378 mmol). Yield: 39 mg (29%). Anal. Calcd. for C_37_H_28_IrN_3_: C, 62.87; H, 3.99; N, 5.94. Found: C, 62.93; H, 3.91; N, 5.62. HRMS (electrospray, *m*/*z*): calcd for C_37_H_28_IrN_3_Na [M + Na]^+^ 730.1807; found, 730.1806. ^1^H NMR (300 MHz, CD_2_Cl_2_): δ 8.14 (d, *J* = 8.2 Hz, 1H), 7.91 (td, *J* = 7.8, 1.9 Hz, 1H), 7.73–7.68 (m, 3H), 7.64 (t, *J* = 7.6 Hz, 1H), 7.58–7.46 (m, 4H), 7.38 (d, *J* = 7.5 Hz, 1H), 7.26 (s, 1H), 7.14 (d, *J* = 6.2 Hz, 1H), 7.01–6.94 (m, 2H), 6.93–6.86 (m, 1H), 6.68 (dd, *J* = 8.6, 7.3 Hz, 2H), 6.55–6.48 (m, 2H), 6.44 (ddd, *J* = 7.0, 5.4, 1.5 Hz, 1H), 6.35–6.29 (m, 1H), 2.79, 2.70 (both s, 3H each, Me). ^13^C{^1^H}-NMR (75 MHz, CD_2_Cl_2_): δ 167.7, 167.6, 165.4, 161.6, 159.5, 158.7 (all C_q_), 154.4, 152.7 (all CH), 152.3, 144.9, 144.1, 143.9 (all C_q_) 142.2, 142.1 (all CH), 139.8 (C_q_), 137.4, 136.2, 135.7, 125.8, 124.8, 124.4, 123.9, 122.6, 122.0 (all CH), 121.9 (2C), 121.6, 121.3 (2C), 121.2, 120.6, 117.4, 115.3 (all CH), 61.3, 61.2 (both C_q_), 24.0, 22.8 (both Me).

## Supporting Information Available

General information for the experimental section, structural analysis, computational details and energies of optimized structures, experimental and computed UV/vis spectra, cyclic voltammograms, frontier orbitals, normalized excitation and emission spectra, and NMR spectra. (PDF)
Cartesian coordinates of the optimized structures. (XYZ)

## Supplementary Material

ic2c04106_si_001.pdf

ic2c04106_si_002.xyz
