# Supplementary material for: Ligand Design and Preparation, Photophysical Properties, and Device Performance of an Encapsulated-Type Pseudo-Tris(heteroleptic) Iridium(III) Emitter
Source: Inorg Chem. 2023 Feb 21;62(9):3847–59. doi: 10.1021/acs.inorgchem.2c04106 (PMC10880055; doi:10.1021/acs.inorgchem.2c04106)
Supplement: Supplementary file 1 — ic2c04106_si_001.pdf [file ic2c04106_si_001.pdf]

Supporting Information for

Ligand Design and Preparation, Photophysical Properties and Device Performance of an Encapsulated-Type *Pseudo*-Tris(heteroleptic) Iridium(III) Emitter

Vadim Adamovich,[b] *Llorenç Benavent,*[a] *Pierre-Luc T. Boudreault,*[b] *Miguel A. Esteruelas,* *[,a] *Ana M. López,*[a] *Enrique Oñate,*[a] *and Jui-Yi Tsai*[b]

[a] Departamento de Química Inorgánica, Instituto de Síntesis Química y Catálisis Homogénea (ISQCH), Centro de Innovación en Química Avanzada (ORFEO-CINQA), Universidad de Zaragoza-CSIC, 50009 Zaragoza, Spain.

[b] Universal Display Corporation, Ewing, New Jersey 08618, United States.

*Corresponding author's e-mail address: maester@unizar.es

CONTENTS

Experimental Section: General Information S3

Structural Analysis of Complexes 9 and 10 S4

Computational Details S5

Energies of Optimized Structures S5

UV-vis Spectra (Observed and Calculated) S8

Analysis of Computed UV/Vis Data S9

Cyclic Voltammograms S11

Theoretical Analysis of Molecular Orbitals S12

Spin Density Distributions S16

Normalized Excitation and Emission Spectra of Complexes S16

NMR Spectra S18

References S35

Experimental Section: General information. All reactions were performed with rigorous exclusion of air at an argon/vacuum manifold using standard Schlenk-tube or glovebox techniques. Solvents were dried by the usual procedures and distilled under argon prior to use or obtained oxygen- and water-free from an MBraun solvent purification apparatus. NMR spectra were recorded on a Bruker ARX 300, Bruker Avance 300 MHz, or a Bruker Avance 400 MHz instruments. Chemical shifts (expressed in parts per million) are referenced to residual solvent peaks (^{1}H, $^{13}C\{^{1}H\}$) or external $CFCl_3$ (^{19}F). Coupling constants *J* are given in Hertz. C, H, and N analyses were carried out in a Perkin-Elmer 2400-B Series II CHNS-Analyzer. High-resolution electrospray mass spectra (HRMS) were acquired using a MicroTOF-Q hybrid quadrupole time-of-flight (Bruker Daltonics, Bremen, Germany). Attenuated total reflection infrared spectra (ATR-IR) of solid samples were run on a Perkin-Elmer Spectrum 100 FT-IR spectrometer. UV-visible spectra were registered on an Evolution 600 spectrophotomer. Steady-state photoluminescence spectra were recorded on a Jobin-Yvon Horiba Fluorolog FL-3-11 spectrofluorimeter. Emission and excitation spectra have been corrected. The detector employed was a TBX-PS detector power source. Lifetime measurements were carried out using HORIBA JOBIN IBON leds of 295 nm for complex **9** and 390 nm for complex **10**, which were the nearest suitable energies to the excitation maxima of the complexes. Emission wavelengths were fixed to the emission maxima for each compound. Data were fitted using HORIBA JOBIN IBON DAS6.v6.1 software. Quantum yields were measured using the Hamamatsu Absolute PL Quantum Yield Measurement System C11347-11. Cyclic voltammetry measurements were performed using a Voltalab PST050 potentiostat with Pt wire as working electrode, Pt wire as counter electrode, and saturated calomel (SCE) as reference electrode. The experiments were carried out under argon in acetonitrile solutions (10^{-3} M), with Bu_4NPF_6 as supporting electrolyte (0.1 M). Scan rate was 100 $mV \cdot s^{-1}$. The potentials were referenced to the ferrocene/ferrocenium (Fc/Fc^{+}) couple. The emitter **10** has been tested in bottom emission OLED structures with the device pixel area 2 mm^2. The glass coated ITO substrates were patterned by photolithography. The device pixel area was defined by the photoresist grid. The devices were fabricated by high vacuum ($<10^{-7}$ Torr) thermal evaporation (VTE). Organic layers have been deposited with the deposition rate 2Å/s. All devices were encapsulated with a glass lid sealed with an epoxy resin in a nitrogen glove box (<1 ppm of H_2O and O_2) immediately after fabrication, and a moisture getter was incorporated inside the package. The HOMO and LUMO energy levels for the materials of the devices were estimated from the oxidation and reduction potentials derived from differential pulsed voltammetry. Solution cyclic voltammetry and differential pulsed voltammetry were performed using a CH Instruments model 6201B potentiostat using anhydrous dimethylformamide solvent and Bu_4NPF_6 as the supporting electrolyte. Glassy carbon, and platinum and silver wires were used as the working, counter, and

reference electrodes, respectively. Electrochemical potentials were referenced to an internal ferrocene-ferrocenium redox couple (Fc/Fc^+) by measuring the peak potential differences from differential pulsed voltammetry. The corresponding highest occupied molecular orbital (HOMO) energy and lowest unoccupied molecular orbital (LUMO) energy were determined by referencing the cationic and anionic redox potentials to ferrocene (4.8 eV vs. vacuum). Devices electroluminescence spectra and luminance were tested using a Spectrophotometer PR-730 at 10 mA cm^{-2}. Current-voltage-luminance (JVL) measurements were performed using calibrated photodiode.

Structural Analysis of Complexes 9 and 10. X-ray data were collected on a APEX D8 Venture Bruker diffractometer (Mo radiation, λ = 0.71073 Å). The crystals were cooled with a nitrogen flow with an Oxford Cryosystems cooler. Data were corrected for absorption by using a multiscan method applied with the SADABS program.[1] The structures were solved by Patterson or direct methods and refined by full-matrix least squares on F^2 with SHELXL2019,[2] including isotropic and subsequently anisotropic displacement parameters. The hydrogen atoms were observed in the last Fourier Maps or calculated, and refined freely or using a restricted riding model.

For **9** we were unable to distinguish between the free phenyl and pyridine groups, so we chose to refine with 50% occupancy for both in the two positions.

Crystal data for **9** (CCDC2215363): $C_{38}H_{29}ClIrN_3O$, 0.5(C_5H_{12}), M_W 807.36, colourless, neddle (0.300 x 0.010 x 0.010 mm^3), triclinic, space group P-1, *a*: 9.3861(5) Å, *b*: 12.8405(7) Å, *c*: 13.8214(8) Å, *α*: 91.545(2)°, *β*: 95.651(2)°, *γ*: 95.758(2)°, V = 1648.18(16) $Å^3$, Z = 2, Z' = 1, D_{calc}: 1.627 g cm^{-3}, F(000): 802, T = 100(2) K, μ 4.169 mm^{-1}. 26399 measured reflections (2θ: 3-51°, ω and φ scans 0.5°), 6133 unique (R_{int} = 0.0555); min./max. transm. Factors 0.757/0.862. Final agreement factors were R^1 = 0.0365 (5635 observed reflections, I > 2σ(I)) and wR^2 = 0.0741; data/restraints/parameters 6133/61/443; GoF = 1.107. Largest peak and hole 1.414 (close to Ir atoms) and -3.854 e/ $Å^3$.

Crystal data for **10** (CCDC2215364): $C_{37}H_{28}IrN_3$, CH_2Cl_2, M_W 904.73, yellow, irregular block (0.150 x 0.120 x 0.025 mm^3), triclinic, space group P-1, *a*: 12.2264(5) Å, *b*: 15.6844(6) Å, *c*: 16.8827(7) Å, *α*: 74.9452(15)°, *β*: 81.0241(15)°, *γ*: 74.7828(14)°, V = 3003.3(2) $Å^3$, Z = 4, Z' = 2, D_{calc}: 1.751 g cm^{-3}, F(000): 1560, T = 100(2) K, μ 4.658 mm^{-1}. 210497 measured reflections (2θ: 3-57°, ω and φ scans 0.5°), 15501 unique (R_{int} = 0.0237); min./max. transm. Factors 0.755/0.862. Final agreement factors were R^1 = 0.0237 (14978 observed reflections, I > 2σ(I)) and wR^2 = 0.0546; data/restraints/parameters 15501/6/789; GoF = 1.021. Largest peak and hole 3.039 (close to Ir atoms) and -2.056 e/ $Å^3$.

Computational Details. All calculations in the mechanistic studies were performed at the DFT level using the B3LYP functional[3] supplemented with the Grimme's dispersion correction D3[4] as implemented in Gaussian09.[5] Ir atoms were described by means of an effective core potential SDD for the inner electron[6] and its associated double-ζ basis set for the outer ones, complemented with a set of f-polarization functions for iridium.[7] The 6-31G** basis set was used for the H, C, N, O and Cl.[8] All minima were verified to have no negative frequencies. The geometries were fully optimized in THF (ε = 7.4257) solvent using the continuum SMD model.[9] We performed TD-DFT calculations at the same level of theory in THF calculating the lowest 50 singlet-singlet excitations at the ground state S_0. It has to be noticed that the singlet-triplet excitations are set to zero due to the neglect of spin-orbit coupling in the TDDFT calculations as implemented in G09. The UV/vis absorption spectra were obtained by using the GaussSum 3 software.[10] The phosphorescence emission compares well with the 0-0 transition calculated taking into account the zero point energies (zpe) of the geometries of both the optimized S_0 and T_1 states in THF.

Energies of Optimized Structure of 9

Complex **9** S_0 (THF)

Zero-point correction=	0.574378 (Hartree/Particle)
Thermal correction to Energy=	0.610382
Thermal correction to Enthalpy=	0.611326
Thermal correction to Gibbs Free Energy=	0.504446
Sum of electronic and zero-point Energies=	-2269.353093
Sum of electronic and thermal Energies=	-2269.317089
Sum of electronic and thermal Enthalpies=	-2269.316145
Sum of electronic and thermal Free Energies=	- 2269.423025

Complex **9** T_1 (THF)

Zero-point correction=	0.570310 (Hartree/Particle)
Thermal correction to Energy=	0.606940
Thermal correction to Enthalpy=	0.607884
Thermal correction to Gibbs Free Energy=	0.498870
Sum of electronic and zero-point Energies=	-2269.262398
Sum of electronic and thermal Energies=	-2269.225769

Sum of electronic and thermal Enthalpies= -2269.224824

Sum of electronic and thermal Free Energies= -2269.333838

Complex **10** S_0 (THF)

Zero-point correction= 0.553230 (Hartree/Particle)

Thermal correction to Energy= 0.583345

Thermal correction to Enthalpy= 0.584290

Thermal correction to Gibbs Free Energy= 0.496240

Sum of electronic and zero-point Energies= -1695.161528

Sum of electronic and thermal Energies= -1695.131413

Sum of electronic and thermal Enthalpies= -1695.130469

Sum of electronic and thermal Free Energies= -1695.218518

Complex **10** T_1 (THF)

Zero-point correction= 0.549396 (Hartree/Particle)

Thermal correction to Energy= 0.580241

Thermal correction to Enthalpy= 0.581185

Thermal correction to Gibbs Free Energy= 0.490439

Sum of electronic and zero-point Energies= -1695.081064

Sum of electronic and thermal Energies= -1695.050219

Sum of electronic and thermal Enthalpies= -1695.049275

Sum of electronic and thermal Free Energies= -1695.140021

Complex **10-b** S_0 (THF)

Zero-point correction= 0.553523 (Hartree/Particle)

Thermal correction to Energy= 0.583519

Thermal correction to Enthalpy= 0.584463

Thermal correction to Gibbs Free Energy= 0.496774

Sum of electronic and zero-point Energies= -1695.153732

Sum of electronic and thermal Energies= -1695.123736

Sum of electronic and thermal Enthalpies= -1695.122792

Sum of electronic and thermal Free Energies= -1695.210481

Complex **10-c** S_0 (THF)

Zero-point correction= 0.553218 (Hartree/Particle)
Thermal correction to Energy= 0.583236
Thermal correction to Enthalpy= 0.584180
Thermal correction to Gibbs Free Energy= 0.496464
Sum of electronic and zero-point Energies= -1695.144841
Sum of electronic and thermal Energies= -1695.114824
Sum of electronic and thermal Enthalpies= -1695.113879
Sum of electronic and thermal Free Energies= -1695.201595

Complex **10-d** S_0 (THF)
Zero-point correction= 0.553174 (Hartree/Particle)
Thermal correction to Energy= 0.583256
Thermal correction to Enthalpy= 0.584201
Thermal correction to Gibbs Free Energy= 0.496220
Sum of electronic and zero-point Energies= -1695.146982
Sum of electronic and thermal Energies= -1695.116899
Sum of electronic and thermal Enthalpies= -1695.115955
Sum of electronic and thermal Free Energies= -1695.203936

Chart S1. Possible Isomers of Complex 10 with Their Relative Energy (kcal·mol^{-1}) in THF.

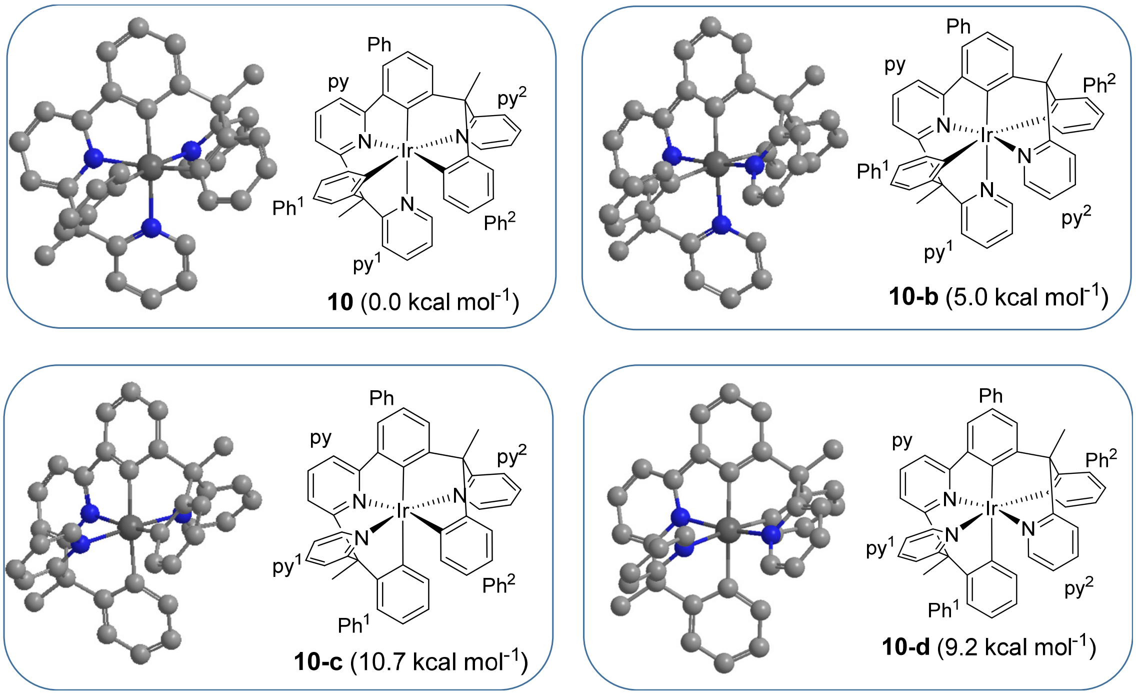

UV-vis Spectra of Complexes 9 and 10 (Observed and Calculated)

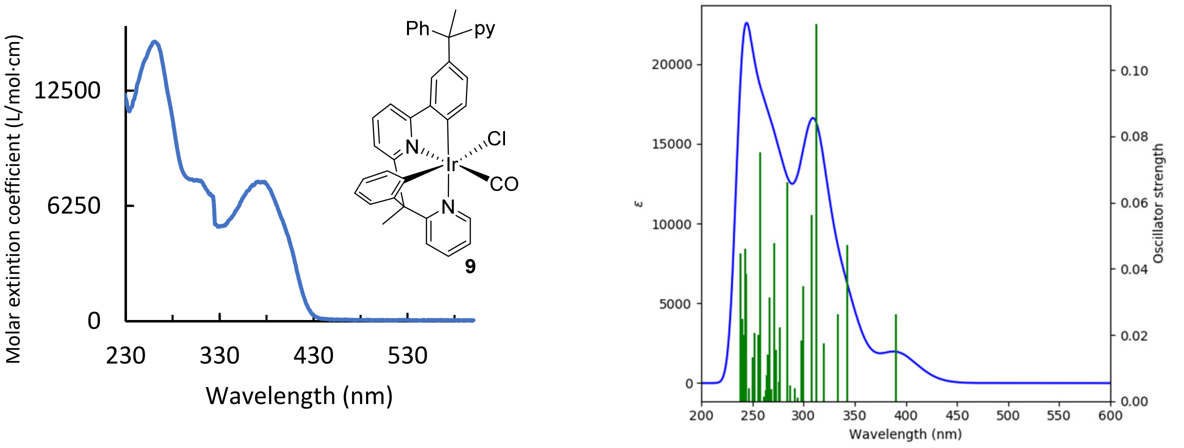

Figure S1. Observed UV-vis spectrum of complex **9** in 2-MeTHF (1.0 x 10^{-4} M) and calculated (B3LYP(GD3)//SDD(f)/6-31G**) in THF.

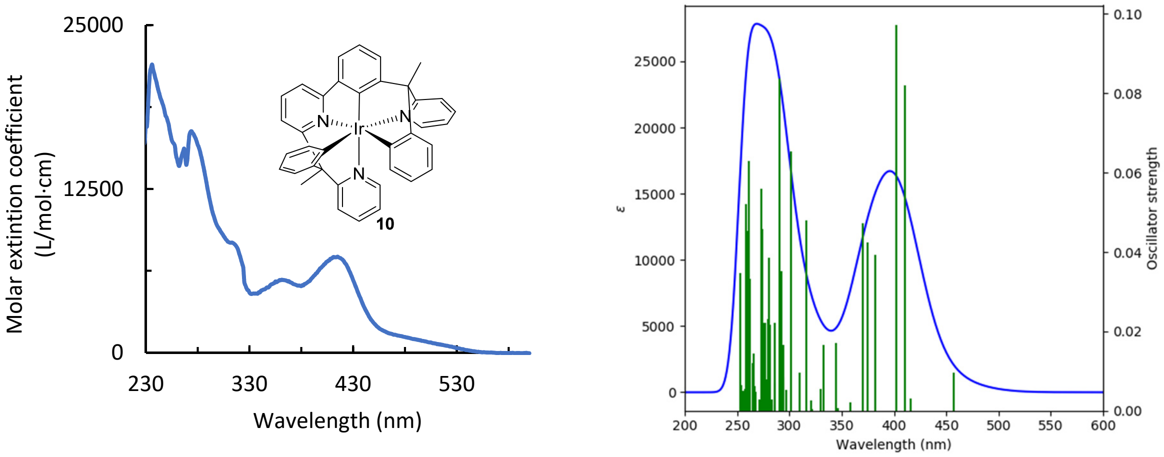

Figure S2. Observed UV-vis spectrum of complex **10** in 2-MeTHF (1.0 x 10^{-4} M) and calculated (B3LYP(GD3)//SDD(f)/6-31G**) in THF.

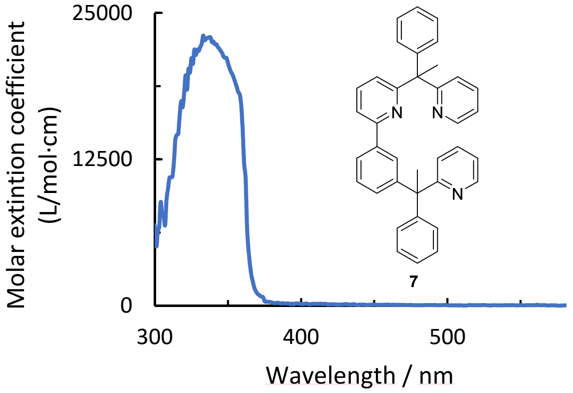

Figure S3. UV-vis spectrum of H_3L (**7**) in 2-MeTHF (1.0 x 10^{-4} M).

Analysis of Computed UV/Vis Data for 9 and 10.

Selected transitions for the calculated UV spectra, energies, oscillator strengths, and molecular orbital contributions to the transitions are given in Tables S1 and S2.

Table S1: Selected transitions for the calculated UV-vis spectrum of 9 in THF

No.	λ (nm)	Osc. Strength	Symmetry	Major contributions	Minor contributions
**1**	452	0	Triplet	HOMO->LUMO (61%), H-1->LUMO (12%)	H-2->LUMO (6%), HOMO->L+1 (4%), H-1->L+1 (2%)
**2**	391	0.0262	Singlet	HOMO->LUMO (97%)	
**7**	342	0.0471	Singlet	H-1->LUMO (93%)	HOMO->L+1 (3%)
**9**	333	0.0263	Singlet	HOMO->L+1 (90%)	H-1->LUMO (3%), H-1->L+1 (2%)
**13**	320	0.0175	Singlet	HOMO->L+2 (87%)	HOMO->L+5 (3%), H-2->LUMO (2%)
**17**	313	0.1140	Singlet	H-2->LUMO (45%), H-3->LUMO (43%)	H-4->LUMO (4%)
**23**	299	0.0348	Singlet	H-1->L+1 (76%)	HOMO->L+3 (9%), H-2->LUMO (3%), HOMO->L+1 (2%)
**35**	284	0.0661	Singlet	H-1->L+2 (80%)	HOMO->L+5 (4%)
**48**	271	0.0476	Singlet	H-2->L+1 (54%), H-3->L+1 (14%), H-4->L+1 (10%)	HOMO->L+4 (9%)
**54**	267	0.0312	Singlet	H-1->L+3 (39%), H-9->LUMO (11%)	H-7->L+1 (8%), H-5->L+1 (6%), H-2->L+2 (6%), H-1->L+5 (6%), H-11->LUMO (5%), H-3->L+2 (3%)

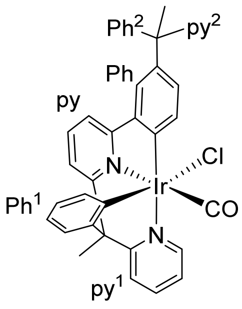

9

Table S2: Selected transitions for the calculated UV-vis spectrum of 10 in THF

No.	λ (nm)	Osc. Strength	Symmetry	Major contributions	Minor contributions
**1**	499	0	Triplet	HOMO->LUMO (83%)	HOMO->LUMO+2 (6%)
**2**	457	0.0095	Singlet	HOMO->LUMO (97%)	
**7**	411	0.0819	Singlet	H-1->LUMO (86%)	HOMO->L+1 (9%), HOMO->L+2 (2%)
**9**	402	0.0973	Singlet	HOMO->L+1 (72%), H-1->LUMO (10%)	HOMO->L+2 (8%), H-1->L+1 (4%), H-1->L+2 (3%)
**12**	382	0.0392	Singlet	H-1->L+1 (91%),	H-1->LUMO (2%), HOMO->L+1 (2%)
**14**	375	0.0424	Singlet	HOMO->L+3 (76%) H-1->L+2 (18%)	
**15**	370	0.0473	Singlet	H-1->L+2 (73%), HOMO->L+3 (20%)	
**53**	290	0.0837	Singlet	H-3->L+2 (62%), H-2->L+2 (19%)	HOMO->L+7 (4%), H-3->L+3 (4%), H-4->L+2 (3%)
**79**	273	0.0560	Singlet	H-5->L+2 (28%), H-5->L+1 (21%), HOMO->L+8 (15%), H-6->L+2 (12%)	H-7->L+1 (6%), H-3->L+3 (3%), H-6->L+1 (2%)
**87**	267	0.0061	Singlet	H-7->L+1 (72%)	H-7->L+2 (9%), H-6->L+1 (3%), H-5->L+2 (2%), H-1->L+7 (2%), HOMO->L+9 (2%)
**94**	260	0.0290	Singlet	H-7->L+2 (41%), H-1->L+8 (20%)	H-7->L+1 (4%), H-6->L+2 (4%), H-6->L+3 (4%), H-4->L+3 (4%), H-1->L+7 (4%)

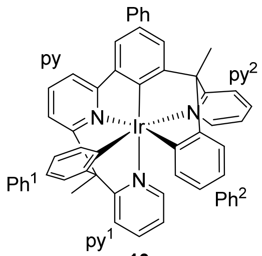

10

Cyclic Voltammograms of Complexes 9 and 10.

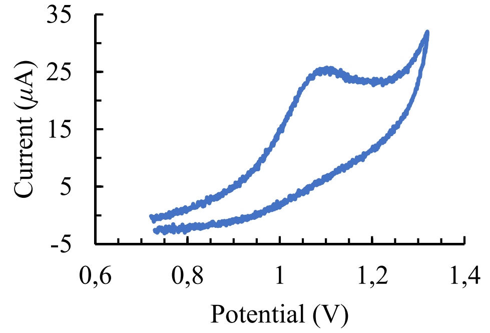

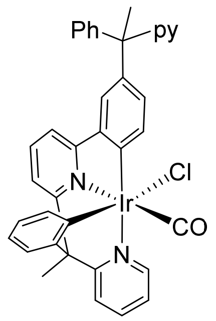

9

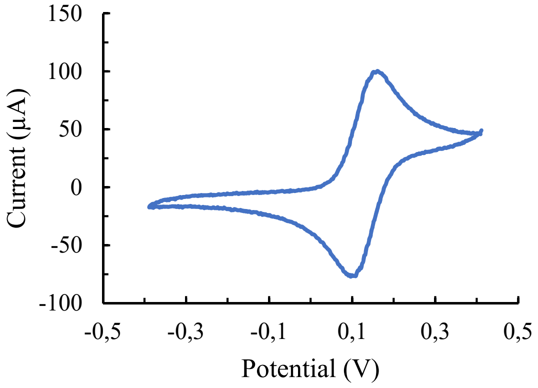

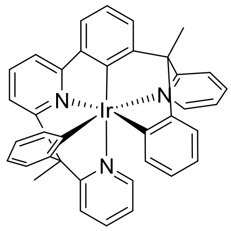

10

Figure S4. Cyclic voltammograms of complexes **9** and **10**, in acetonitrile (10^{-3} M) solutions with Bu_4NPF_6 as supporting electrolyte (0.1 M) at a scan rate of 100 mV s^{-1}. The potentials are referenced to the ferrocene/ferrocenium (Fc/Fc^+) couple.

.

Theoretical Analysis of Molecular Orbitals of Complexes 9 and 10.

Energies and population analysis (%) of molecular orbitals are given in Tables S3 and S4 whereas Figures S4–S6 collects the molecular orbitals.

Table S3: Composition of molecular orbitals of complex 9

MO	eV	Iridium	py	Ph	Ph1	py1	CMe(Ph^2)(py^2)	CMe^1	CO	Cl
L+9	0.08	2	0	3	3	0	91	0	0	0
L+8	-0.03	29	2	8	34	5	10	0	12	0
L+7	-0.17	2	1	2	1	1	94	0	1	0
L+6	-0.26	34	4	2	11	7	2	0	41	0
L+5	-0.36	30	6	2	5	11	2	0	40	3
L+4	-0.56	1	6	3	2	1	87	0	1	0
L+3	-0.73	5	8	2	12	62	4	1	6	0
L+2	-1.08	3	56	11	2	22	2	1	3	0
L+1	-1.30	2	14	9	1	70	1	1	0	0
LUMO	-1.74	5	57	17	0	11	1	1	5	2
HOMO	-5.64	29	6	41	13	1	6	0	0	3
H-1	-6.05	12	10	22	36	1	3	0	1	15
H-2	-6.32	2	15	47	8	0	25	0	0	3
H-3	-6.47	18	1	3	2	0	28	0	2	45
H-4	-6.58	3	1	14	4	0	56	0	0	21
H-5	-6.62	1	2	1	84	5	3	1	0	2
H-6	-6.68	0	1	3	2	0	93	0	0	1
H-7	-6.71	8	6	3	23	8	0	0	2	50
H-8	-6.82	0	2	2	1	0	93	0	0	2
H-9	-6.85	1	0	4	2	0	87	0	0	5

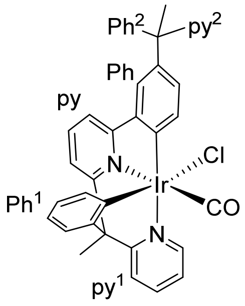

9

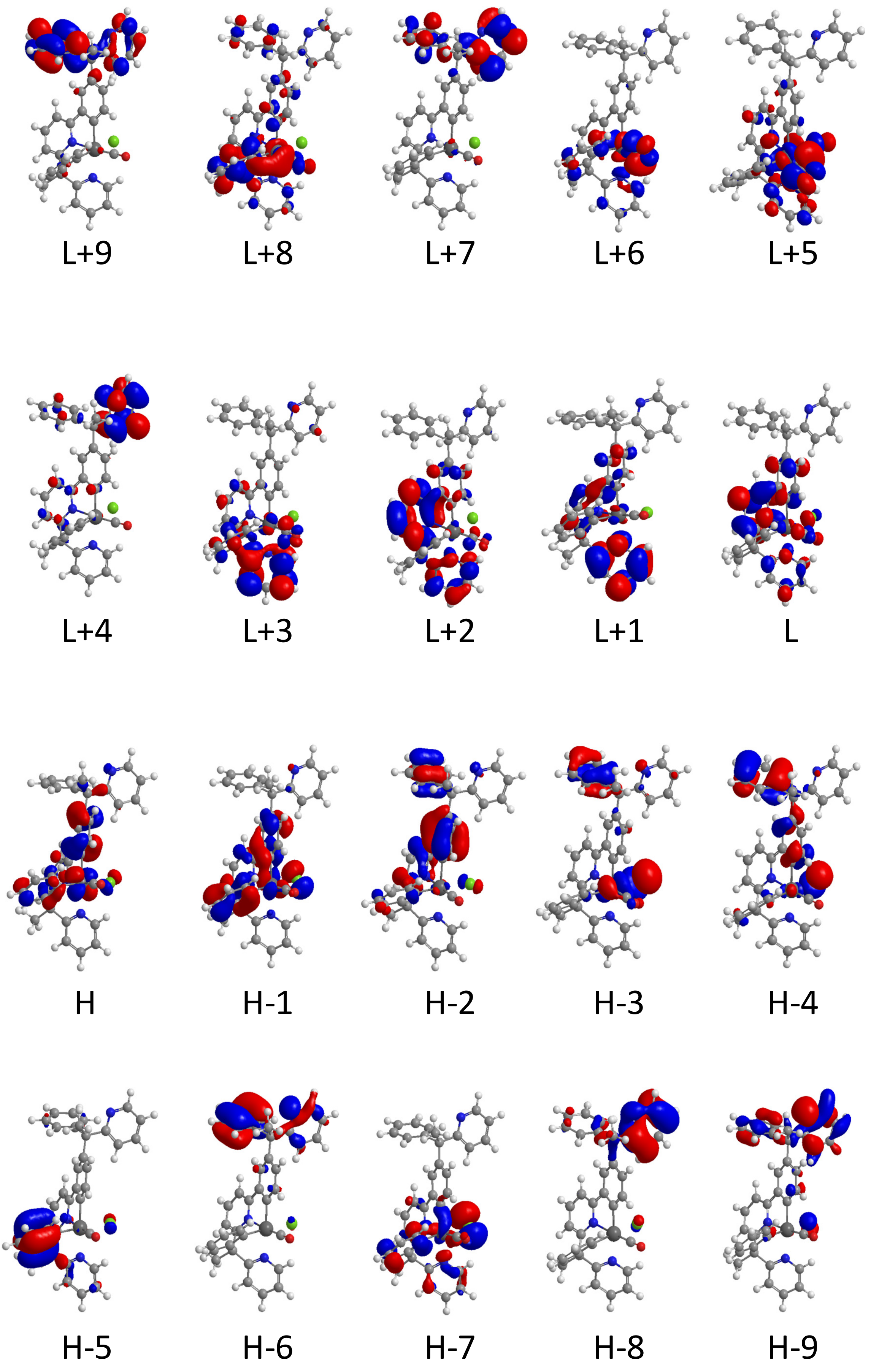

Figure S5. Molecular orbitals of complex **9** (isovalue 0.03 au).

Table S4: Composition of molecular orbitals of 10.

MO	eV	Iridium	Ph	py	Ph^1	py^1	Ph^2	py^2	CMe^1
**L+9**	0.73	7	3	3	36	1	47	1	2
**L+8**	0.45	6	46	3	35	2	1	6	1
**L+7**	0.34	5	20	5	30	2	35	2	1
**L+6**	0.17	95	0	0	2	0	2	1	0
**L+5**	-0.3	3	2	6	4	28	7	50	1
**L+4**	-0.6	3	1	3	3	59	1	29	1
**L+3**	-0.72	4	8	67	5	9	1	6	1
**L+2**	-1	5	10	17	1	4	1	61	1
**L+1**	-1.04	3	8	7	1	62	1	17	1
**LUMO**	-1.29	3	17	45	1	20	2	12	1
**HOMO**	-4.75	46	21	4	8	6	14	1	0
**H-1**	-4.99	45	10	6	13	1	22	3	0
**H-2**	-5.71	52	15	5	17	1	8	2	0
**H-3**	-5.87	25	27	19	18	1	9	1	0
**H-4**	-5.99	11	37	5	15	1	26	3	1
**H-5**	-6.08	3	13	5	27	1	47	2	1
**H-6**	-6.16	10	4	14	28	2	35	6	1
**H-7**	-6.3	4	2	5	58	2	25	3	1
**H-8**	-6.79	19	32	4	7	6	28	2	2
**H-9**	-6.9	23	12	5	25	8	9	17	1

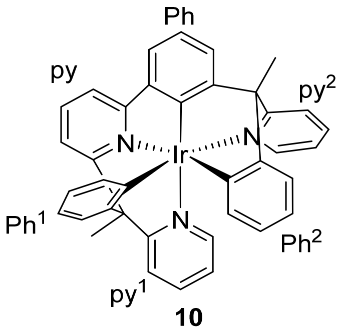

10

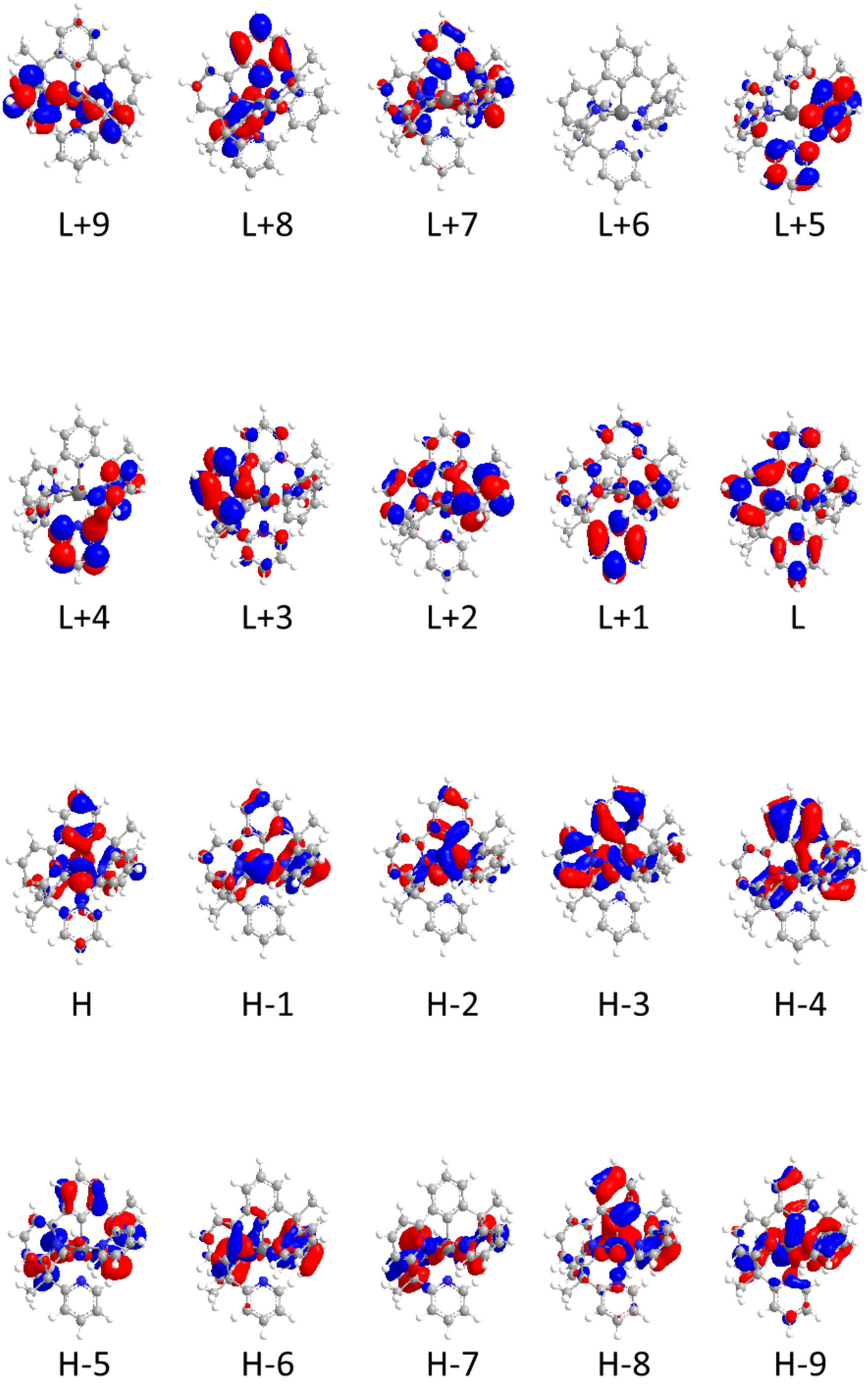

Figure S6. Molecular orbitals of complex **10** (isovalue 0.03 au).

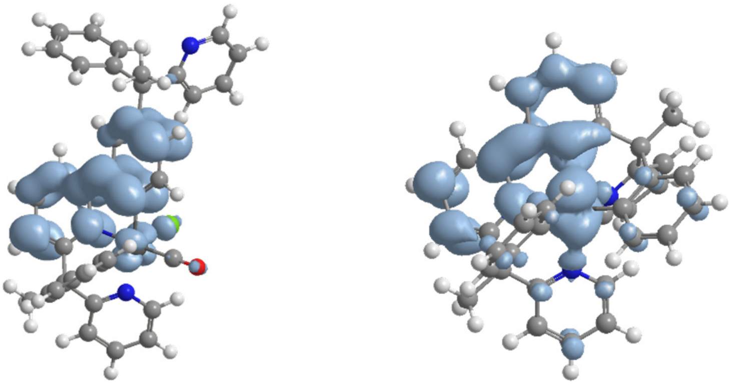

9 **10**

Figure S7. Spin density distributions for the optimized triplet T_1 of complexes **9** and **10** (0.002 isovalue).

Normalized Excitation and Emission Spectra of Complexes 9 and 10.

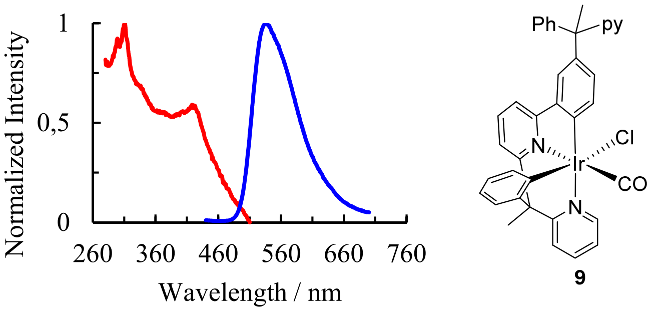

Figure S8. Normalized excitation (λ_{em} = 529 nm; red line) and emission (λ_{exc} = 410 nm; blue line) spectrum **9** in PMMA film (5 wt%) at 298 K.

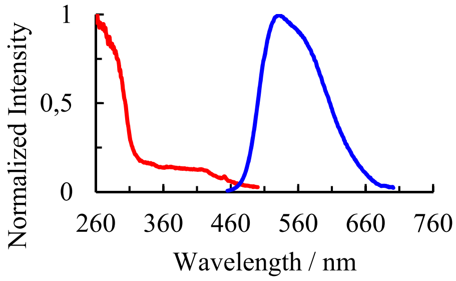

Figure S9. Normalized excitation (λ_{em} = 535 nm; red line) and emission (λ_{exc} = 410 nm; blue line) spectrum of **9** in a 1.0 x 10^{-5} M solution in 2-MeTHF at 298 K.

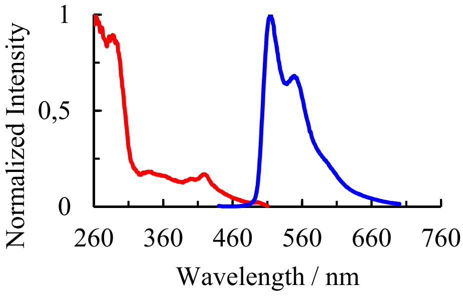

Figure S10. Normalized excitation (λ_{em} = 515 nm; red line) and emission (λ_{exc} = 400 nm; blue line) spectrum of **9** in a 1.0 x 10^{-5} M solution in 2-MeTHF at 77 K.

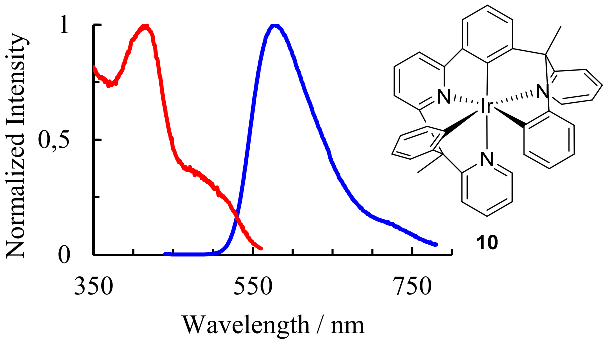

Figure S11. Normalized excitation (λ_{em} = 576 nm; red line) and emission (λ_{exc} = 490 nm; blue line) spectrum of **10** in PMMA film (5 wt%) at 298 K.

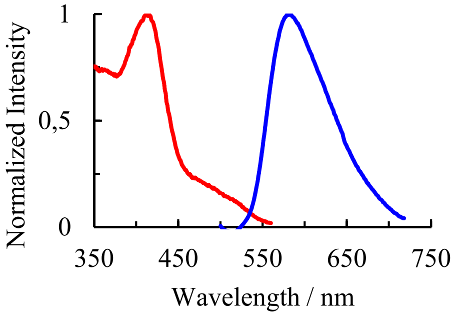

Figure S12. Normalized excitation (λ_{em} = 581 nm; red line) and emission (λ_{exc} = 490 nm; blue line) spectrum of **10** in a 1.0 x 10^{-5} M solution in 2-MeTHF at 298 K.

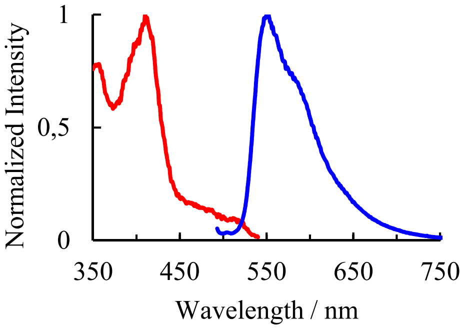

Figure S13. Normalized excitation (λ_{em} = 552 nm; red line) and emission (λ_{exc} = 460 nm; blue line) spectrum of **10** in a 1.0 x 10^{-5} M solution in 2-MeTHF at 77 K.

.

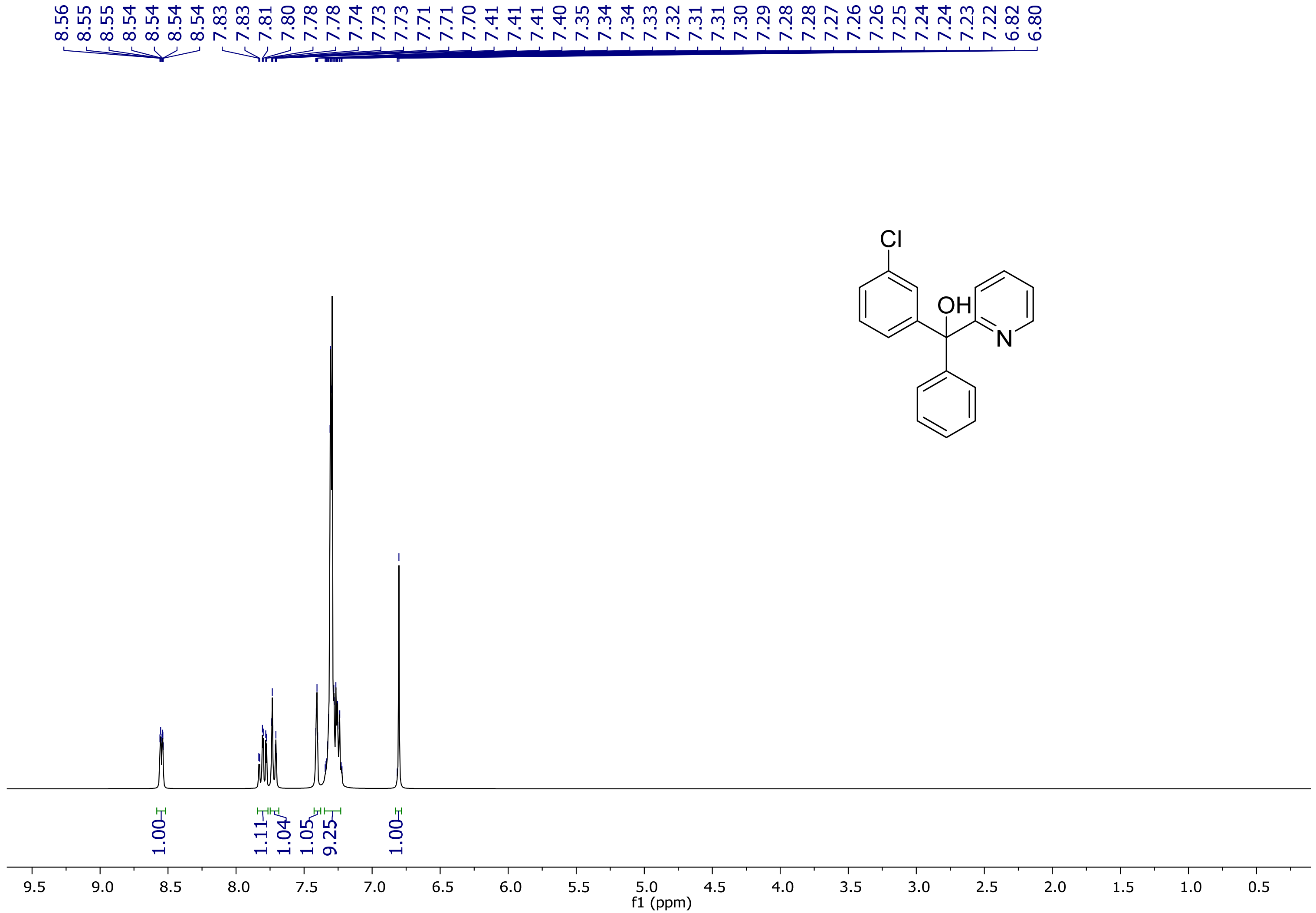

Figure S14. ^{1}H NMR (300 MHz, DMSO-*d*$_6$, 298 K) spectrum of **2**.

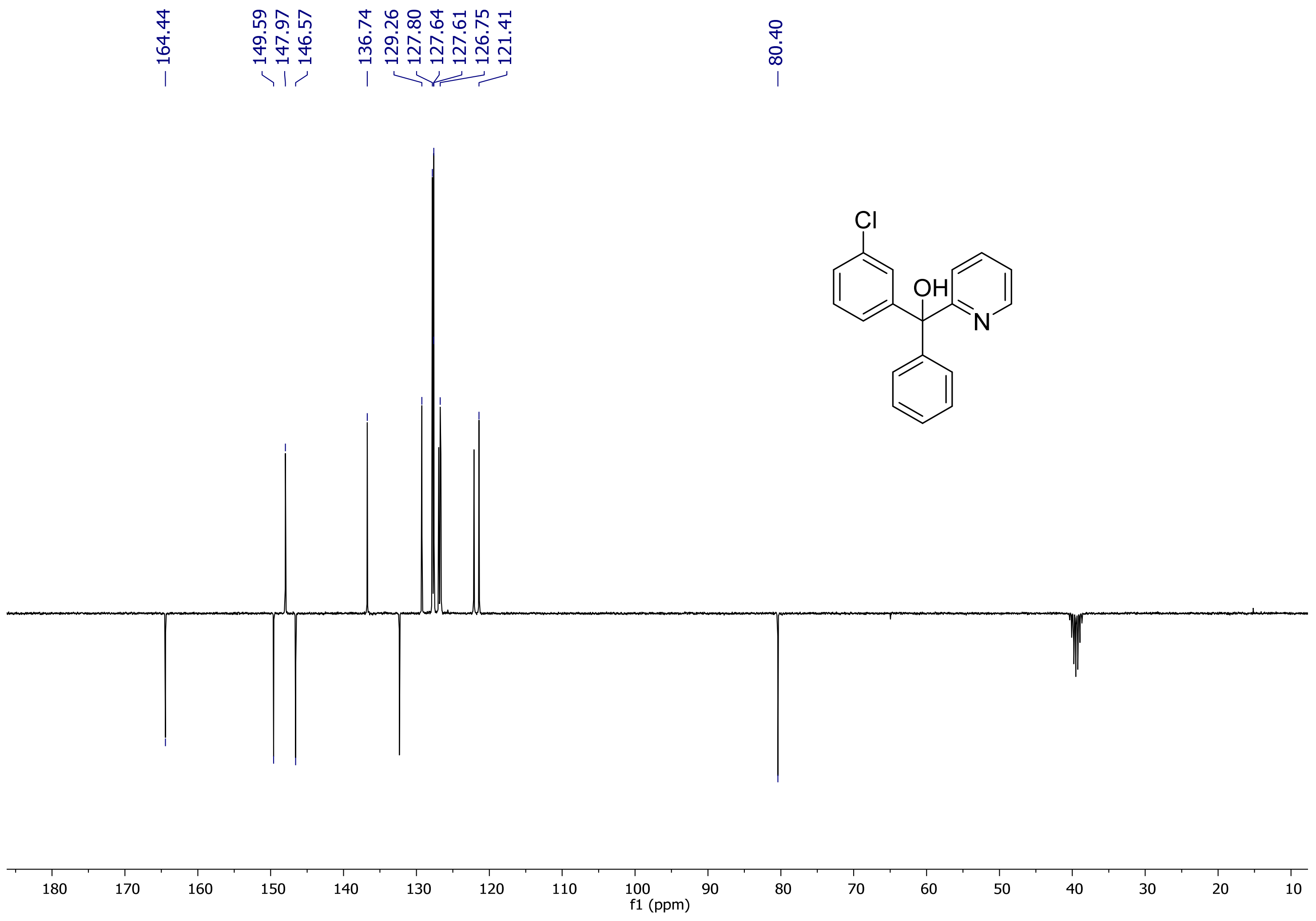

Figure S15. $^{13}C\{^{1}H\}$-APT NMR (75 MHz, DMSO-d_6, 298 K) spectrum of **2**.

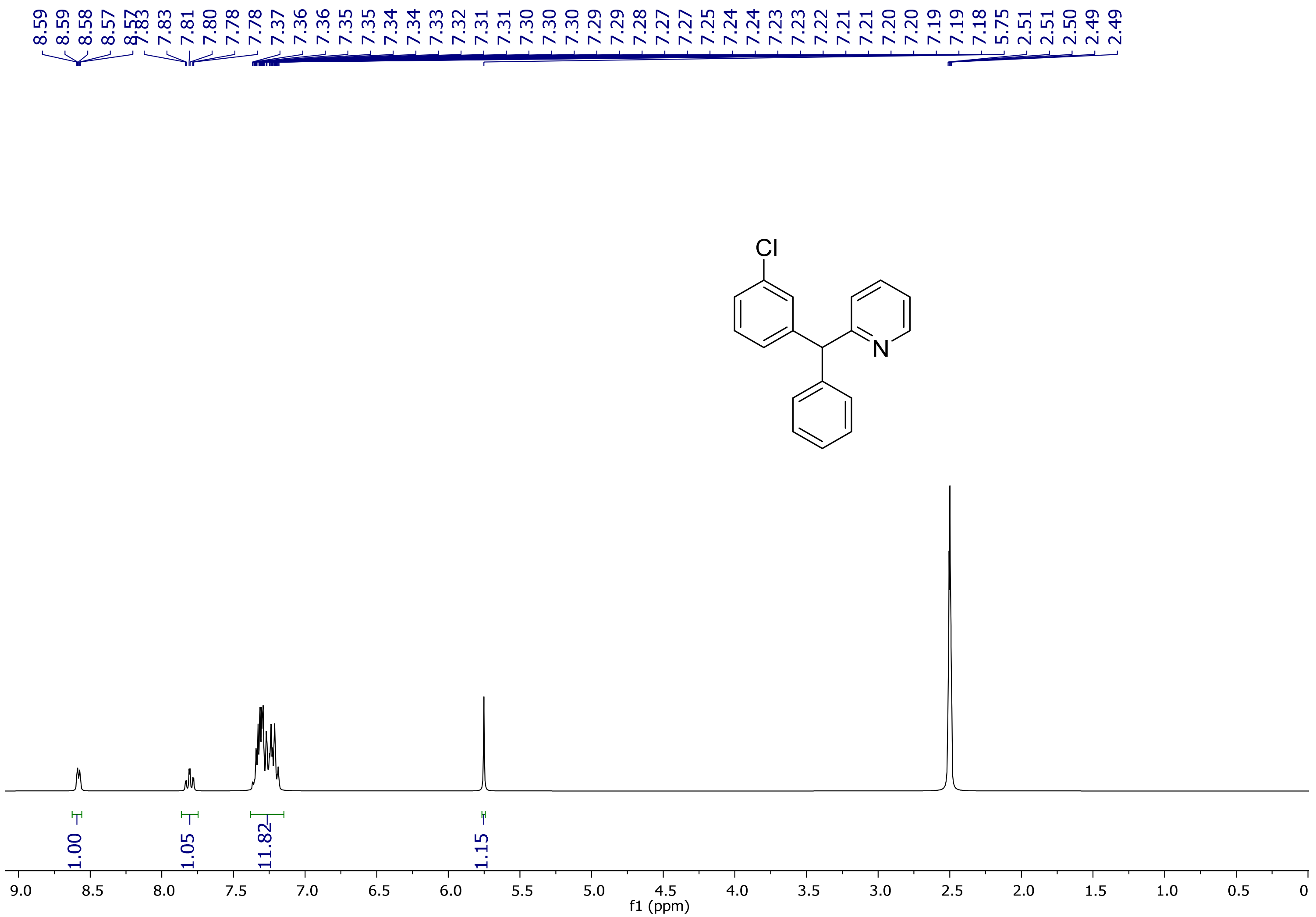

Figure S16. ^{1}H NMR (300 MHz, DMSO-d_6, 298 K) spectrum of **3**.

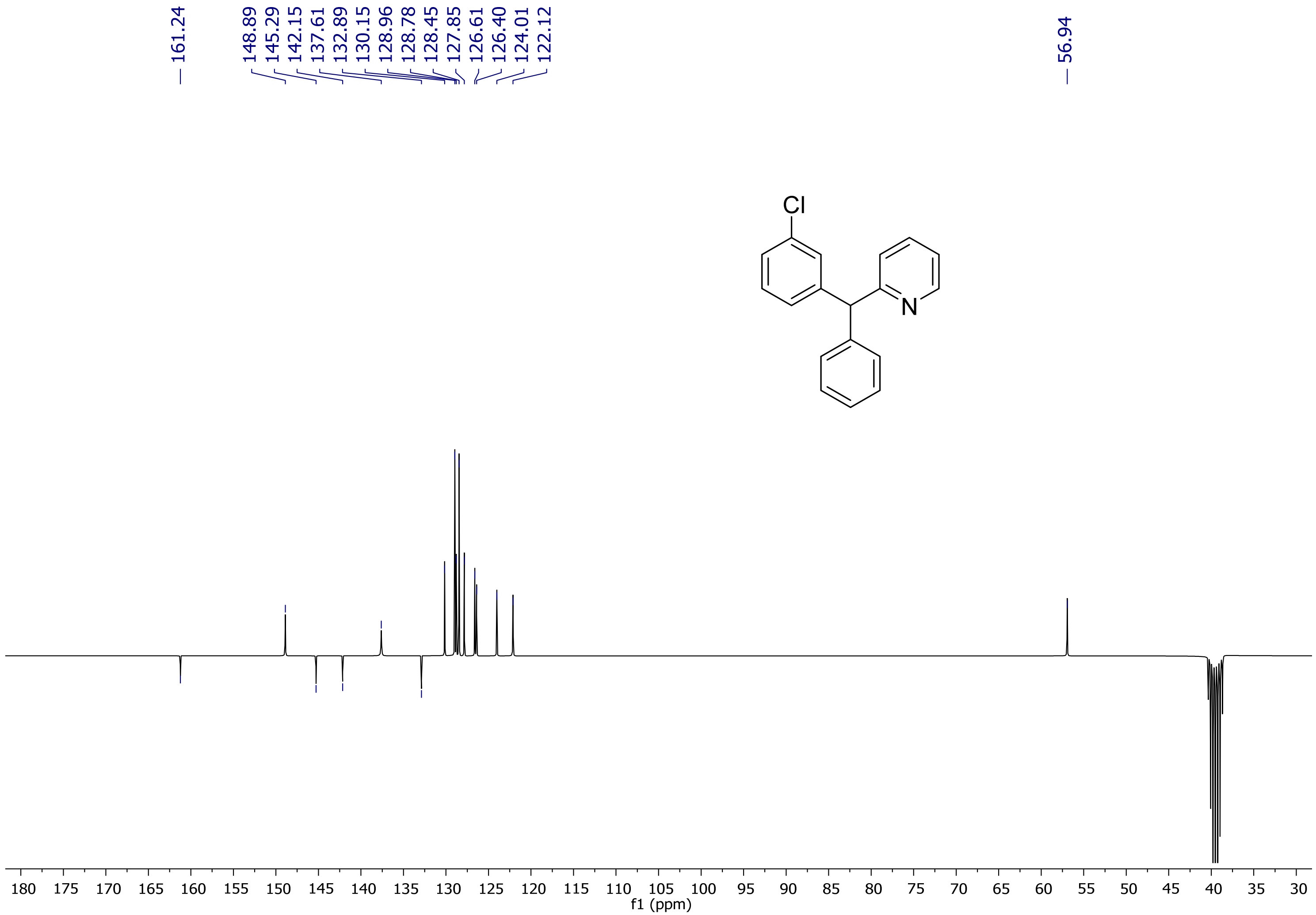

Figure S17. $^{13}C\{^{1}H\}$-APT NMR (75 MHz, DMSO-d_6, 298 K) spectrum of **3**.

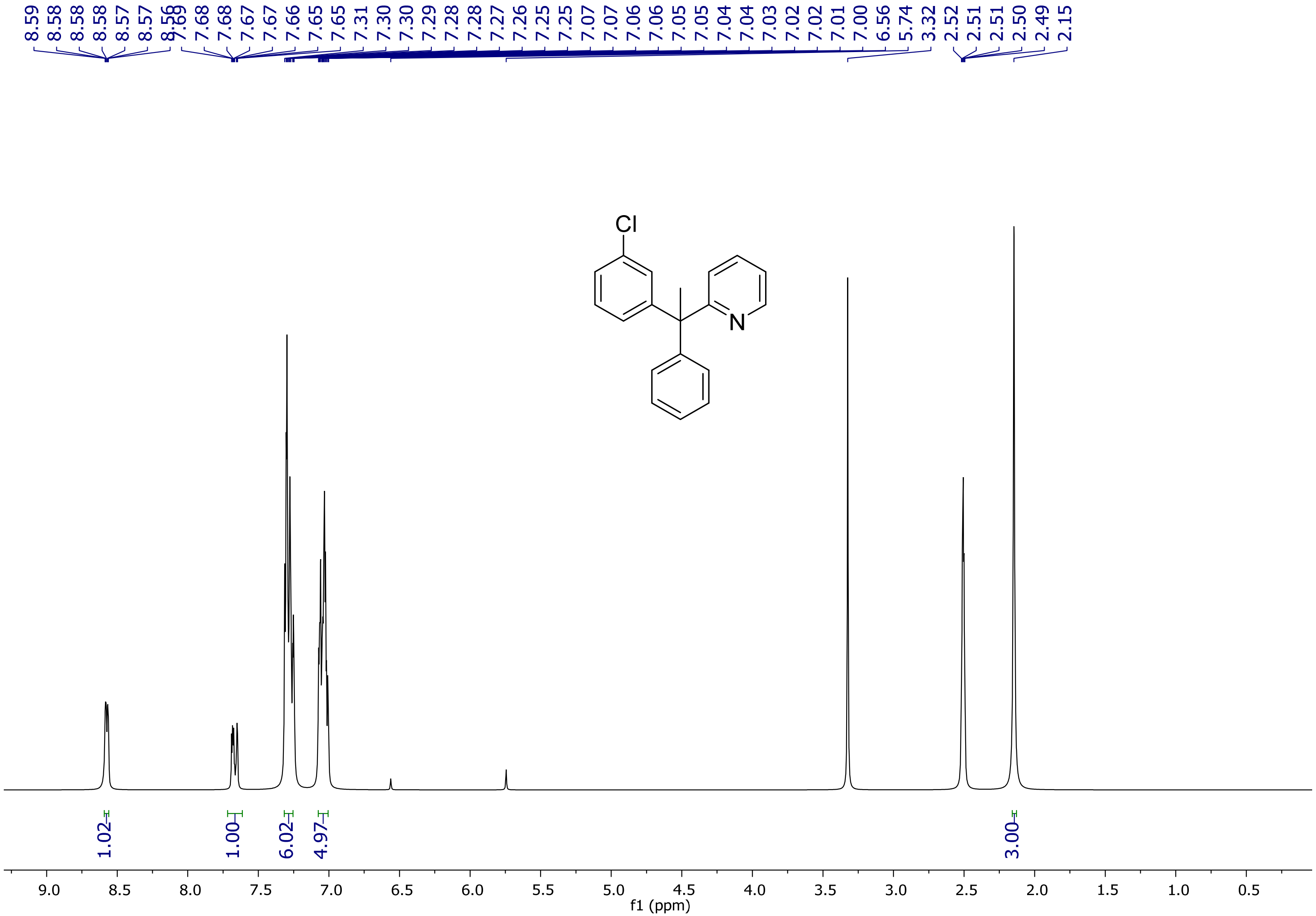

Figure S18. ^{1}H NMR (300 MHz, DMSO-d_6, 298 K) spectrum of **4**.

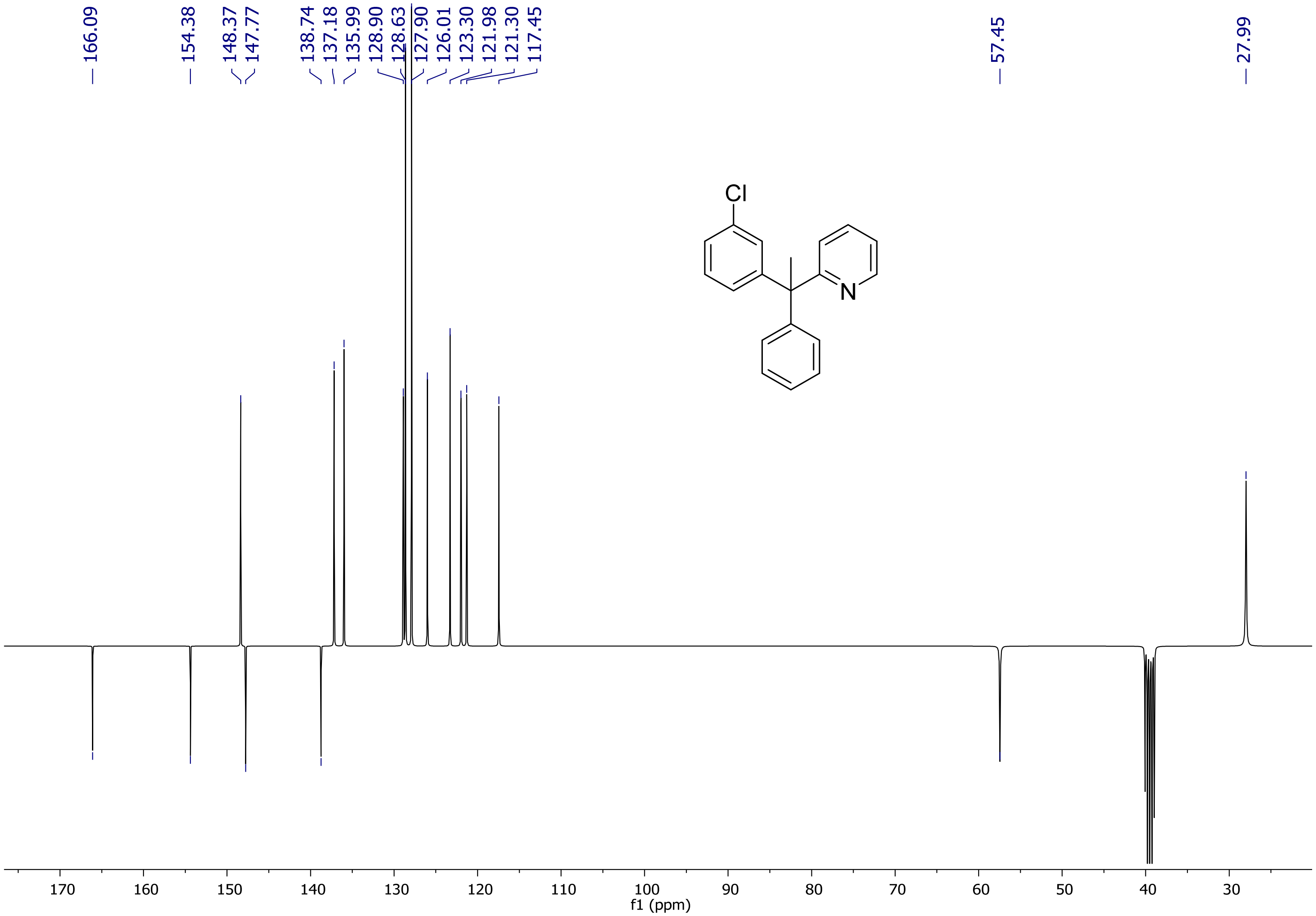

Figure S19. $^{13}C\{^{1}H\}$-APT NMR (75 MHz, DMSO-d_6, 298 K) spectrum of **4**.

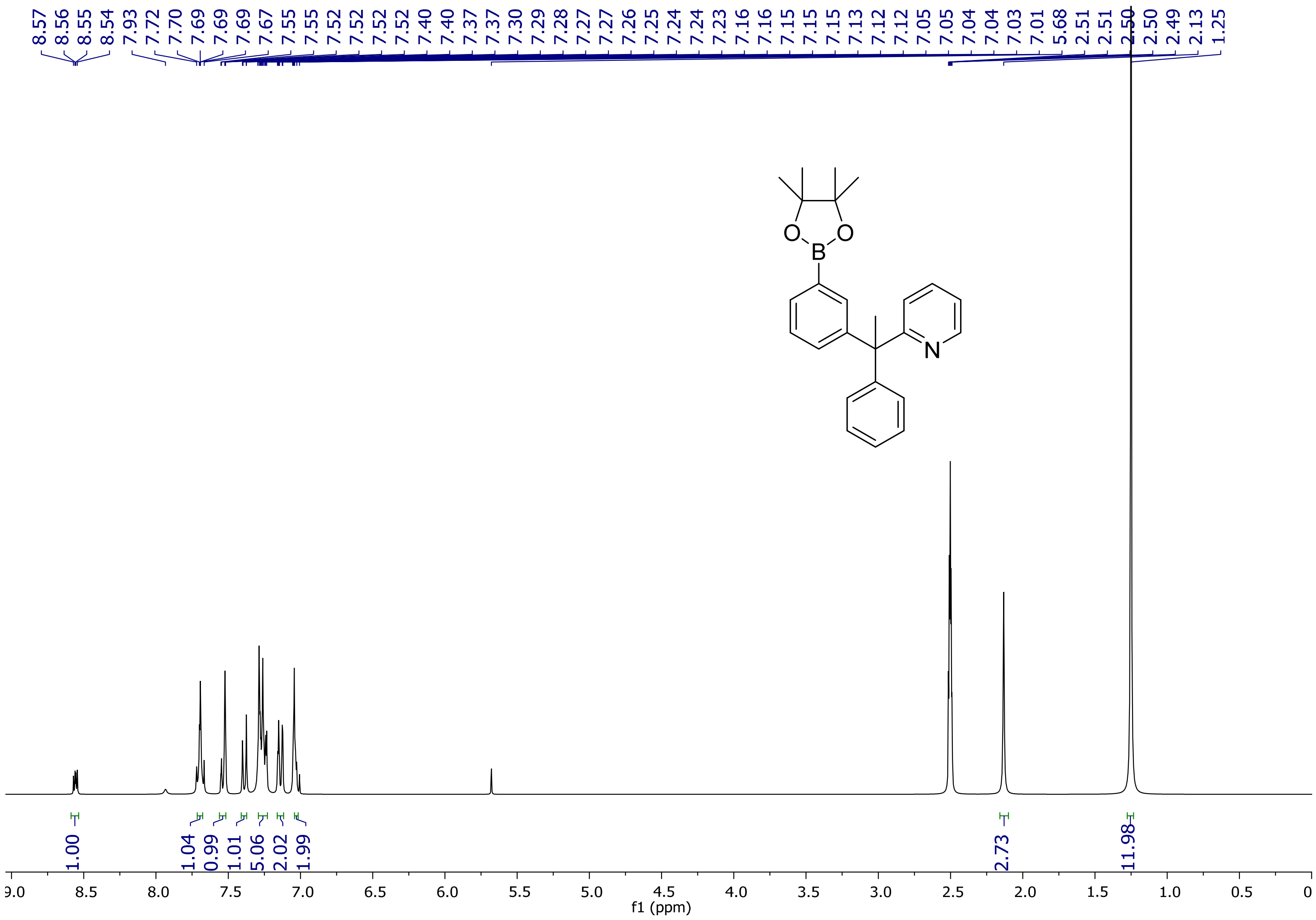

Figure S20. ^{1}H NMR (300 MHz, DMSO-d_6, 298 K) spectrum of **5**.

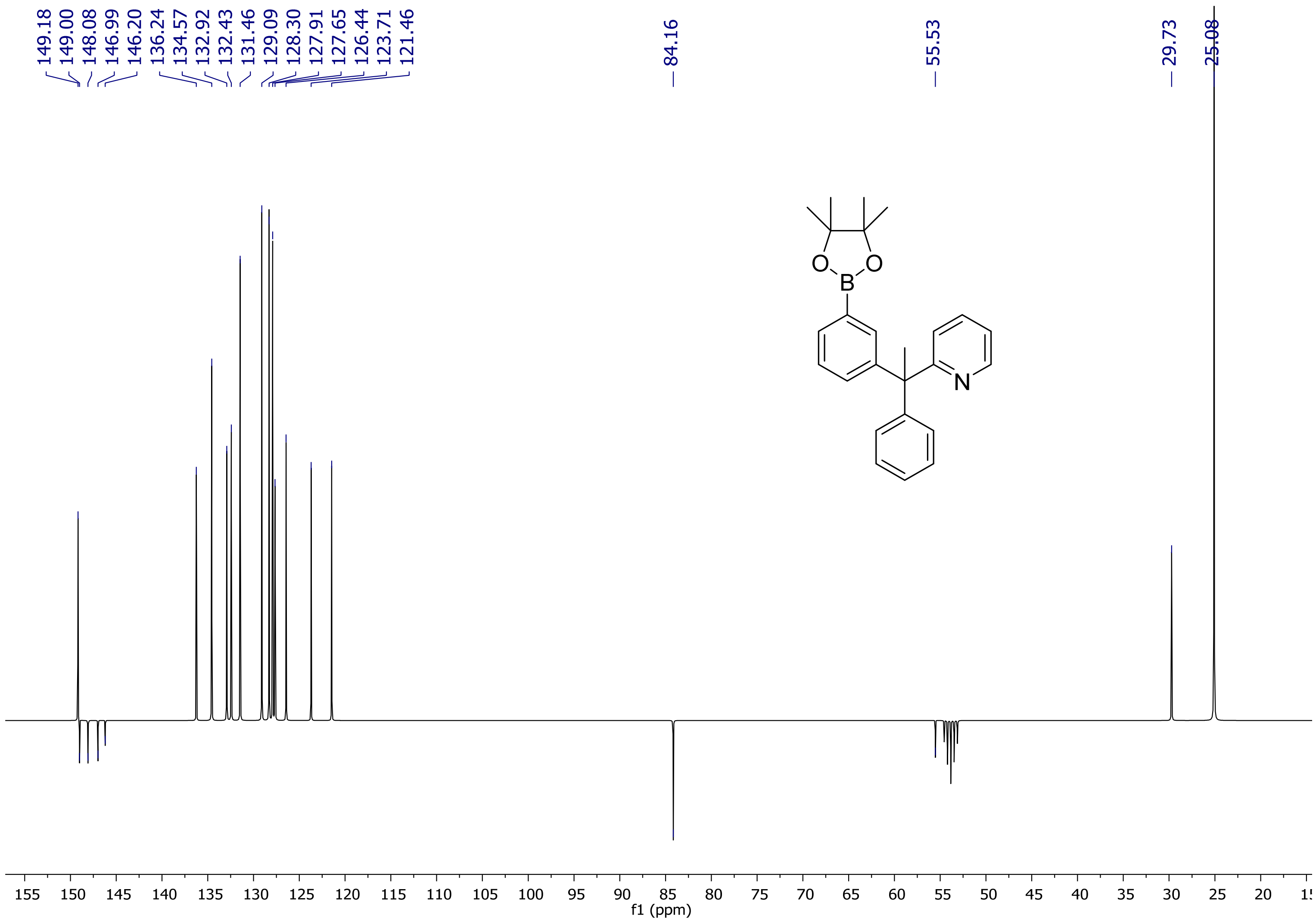

Figure S21. $^{13}C\{^{1}H\}$-APT NMR (75 MHz, CD_2Cl_2, 298 K) spectrum of **5**.

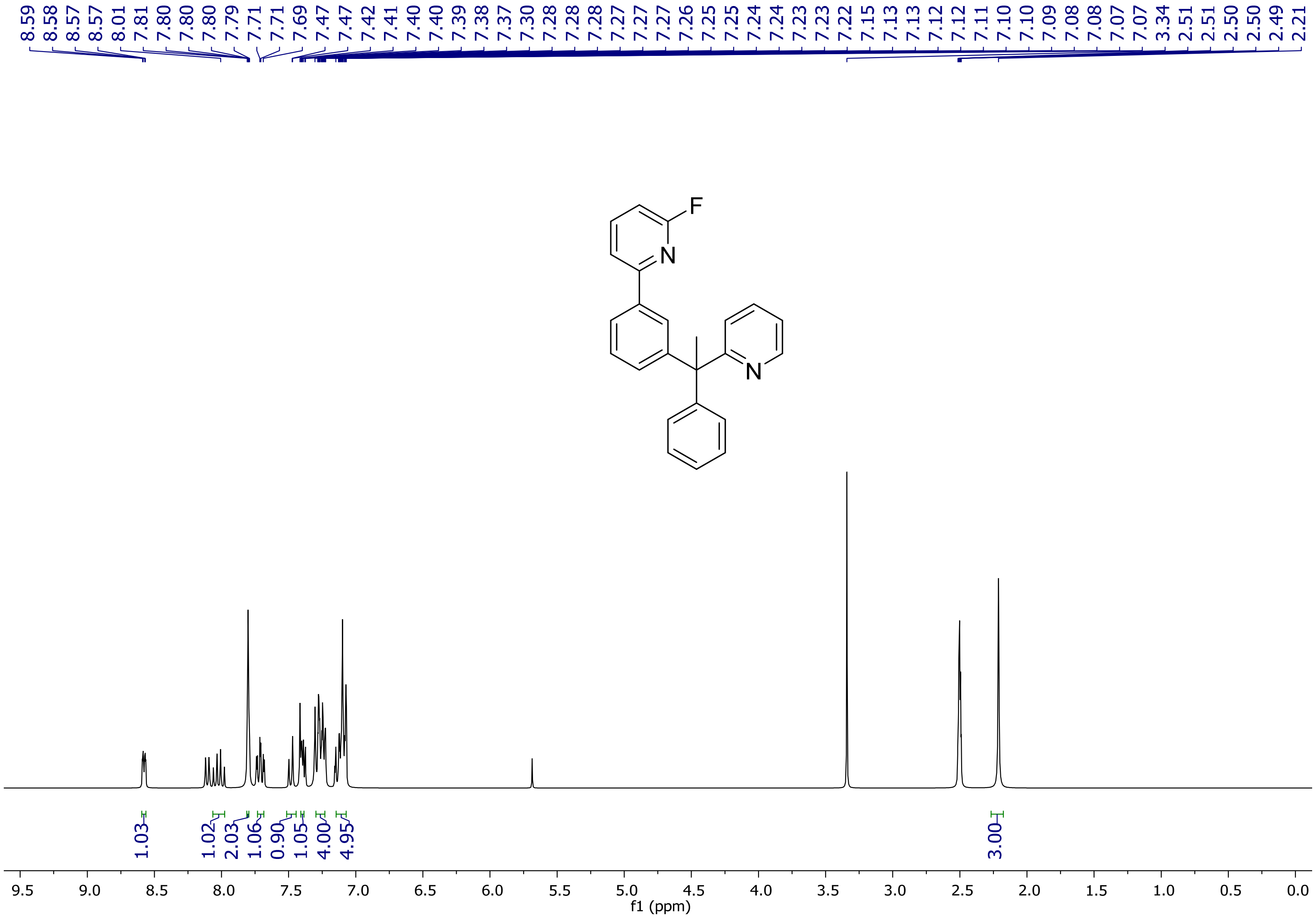

Figure S22. ^{1}H NMR (300 MHz, DMSO-*d*6, 298 K) spectrum of **6**.

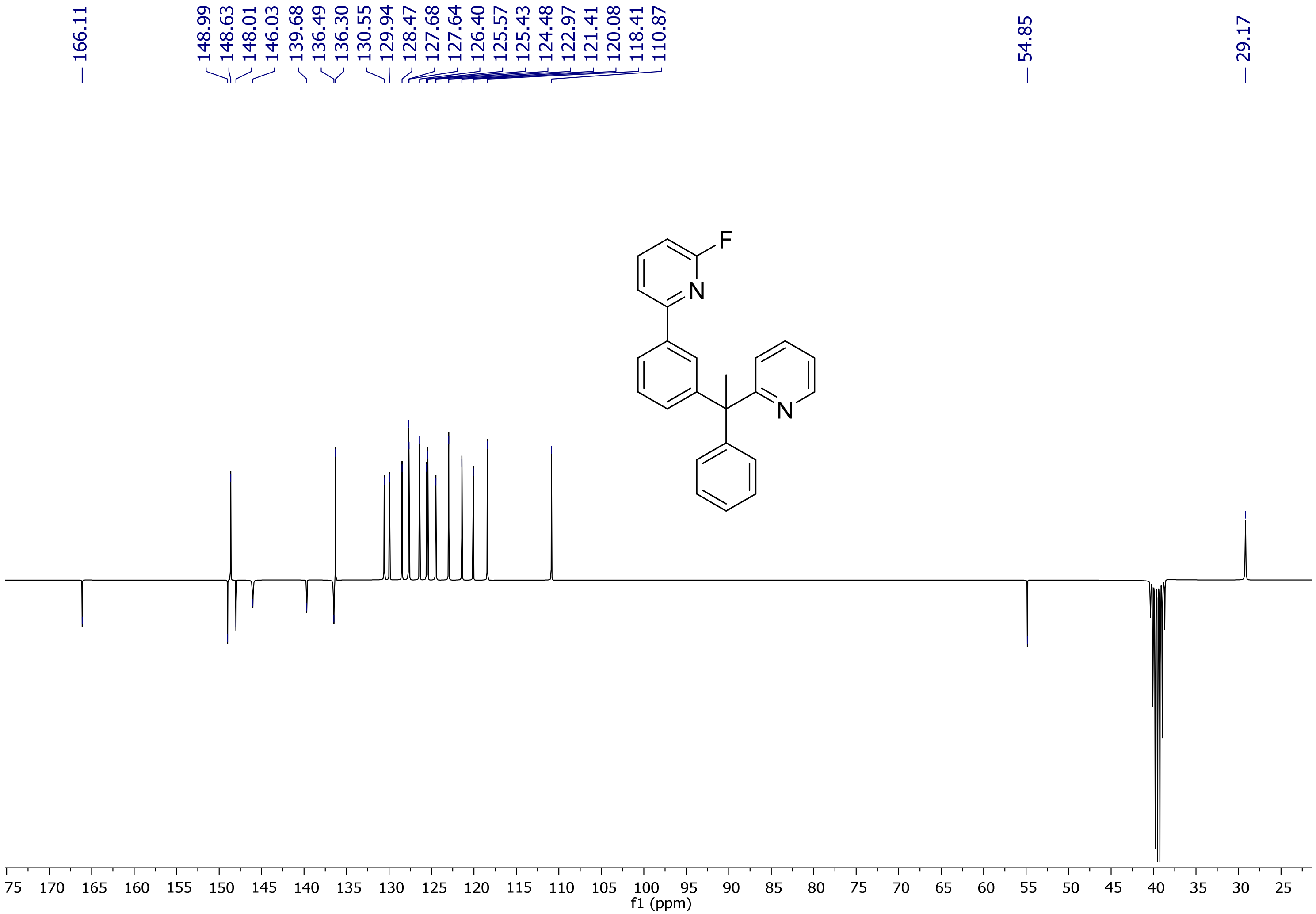

Figure S23. $^{13}C\{^{1}H\}$-APT NMR (75 MHz, DMSO-d_6, 298 K) spectrum of **6**.

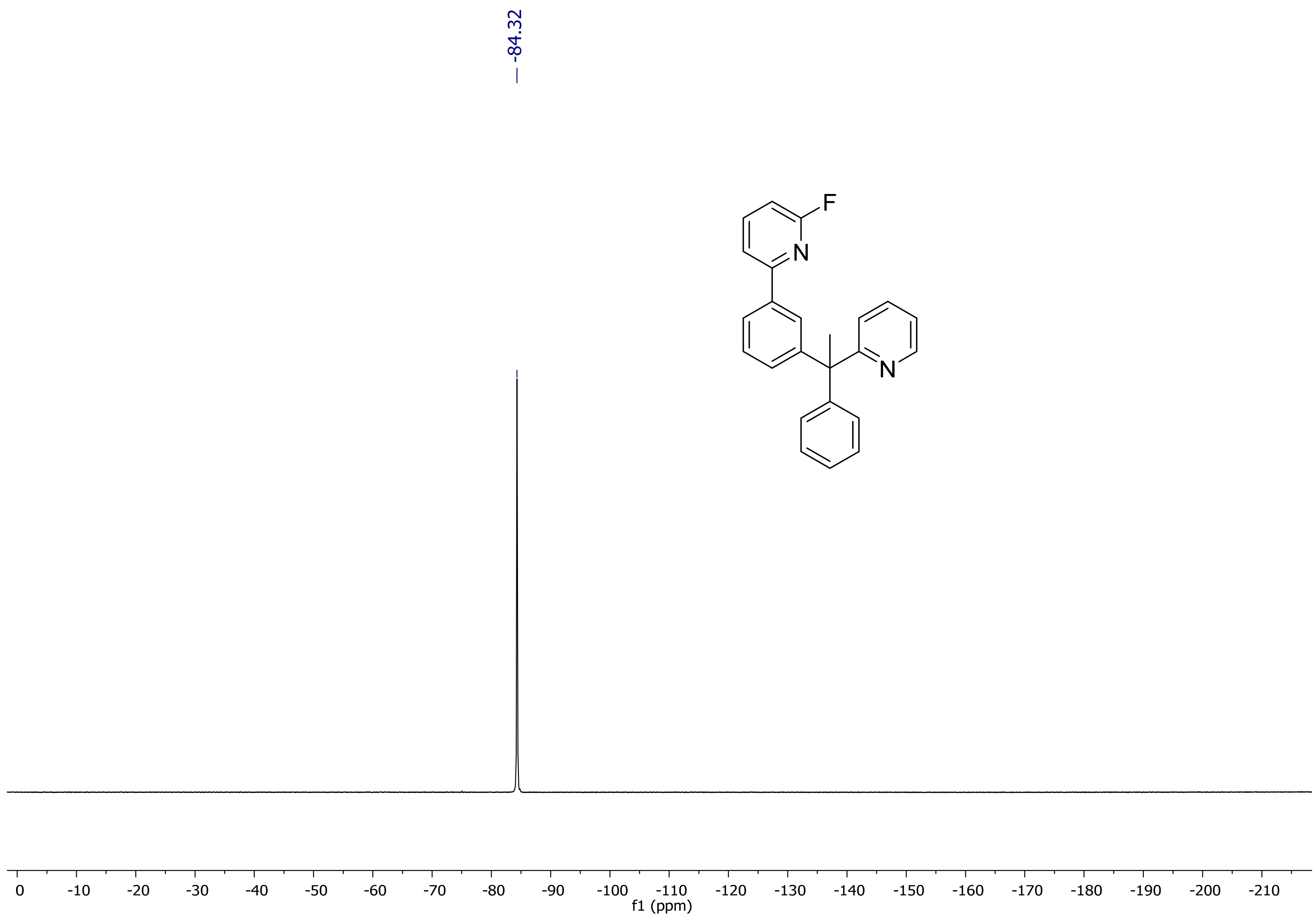

Figure S24. ^{19}F NMR (300 MHz, CD_2Cl_2, 298 K) spectrum of **6**.

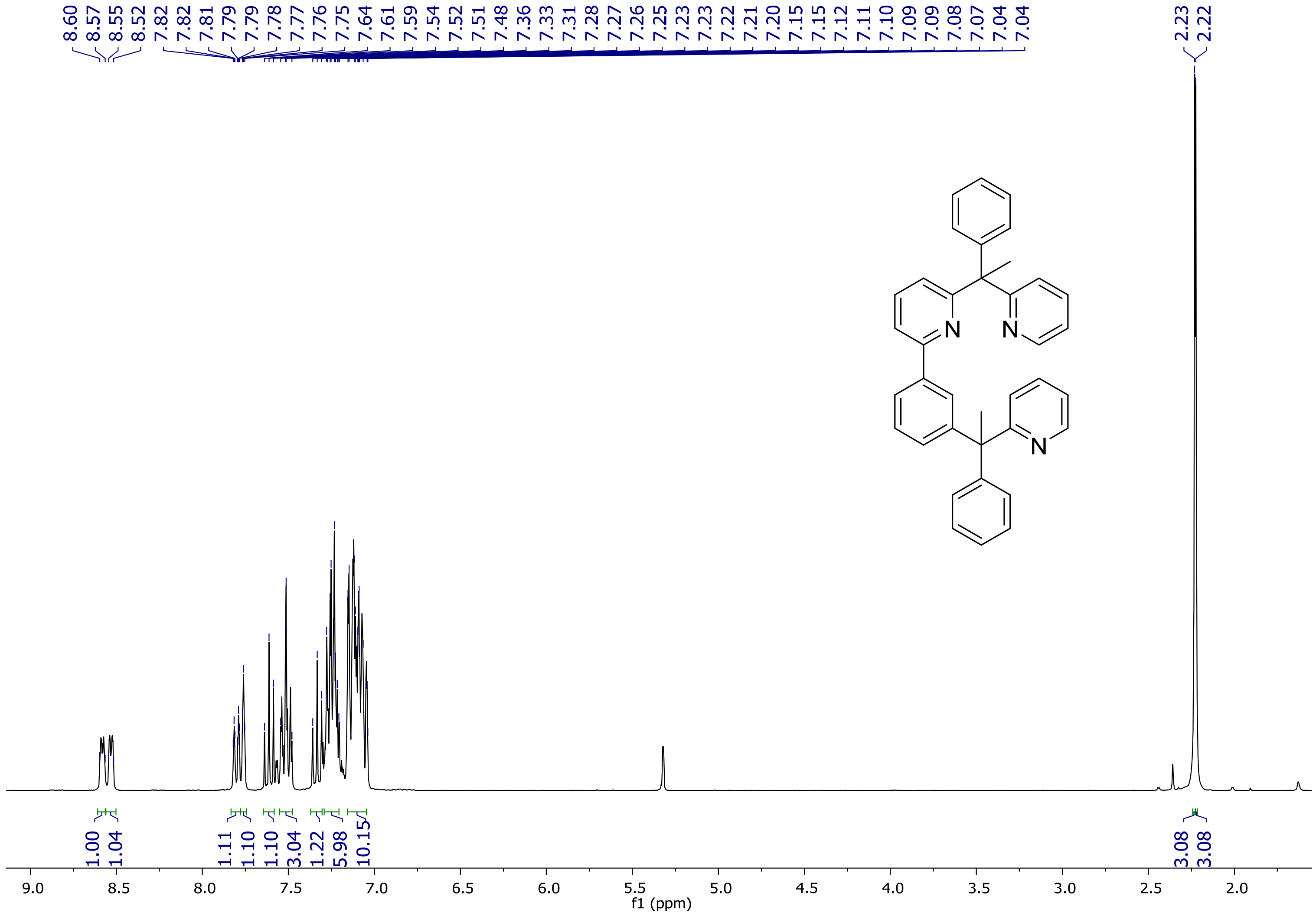

Figure S25. ^{1}H NMR (300 MHz, CD_2Cl_2, 298 K) spectrum of **H_3L** (**7**).

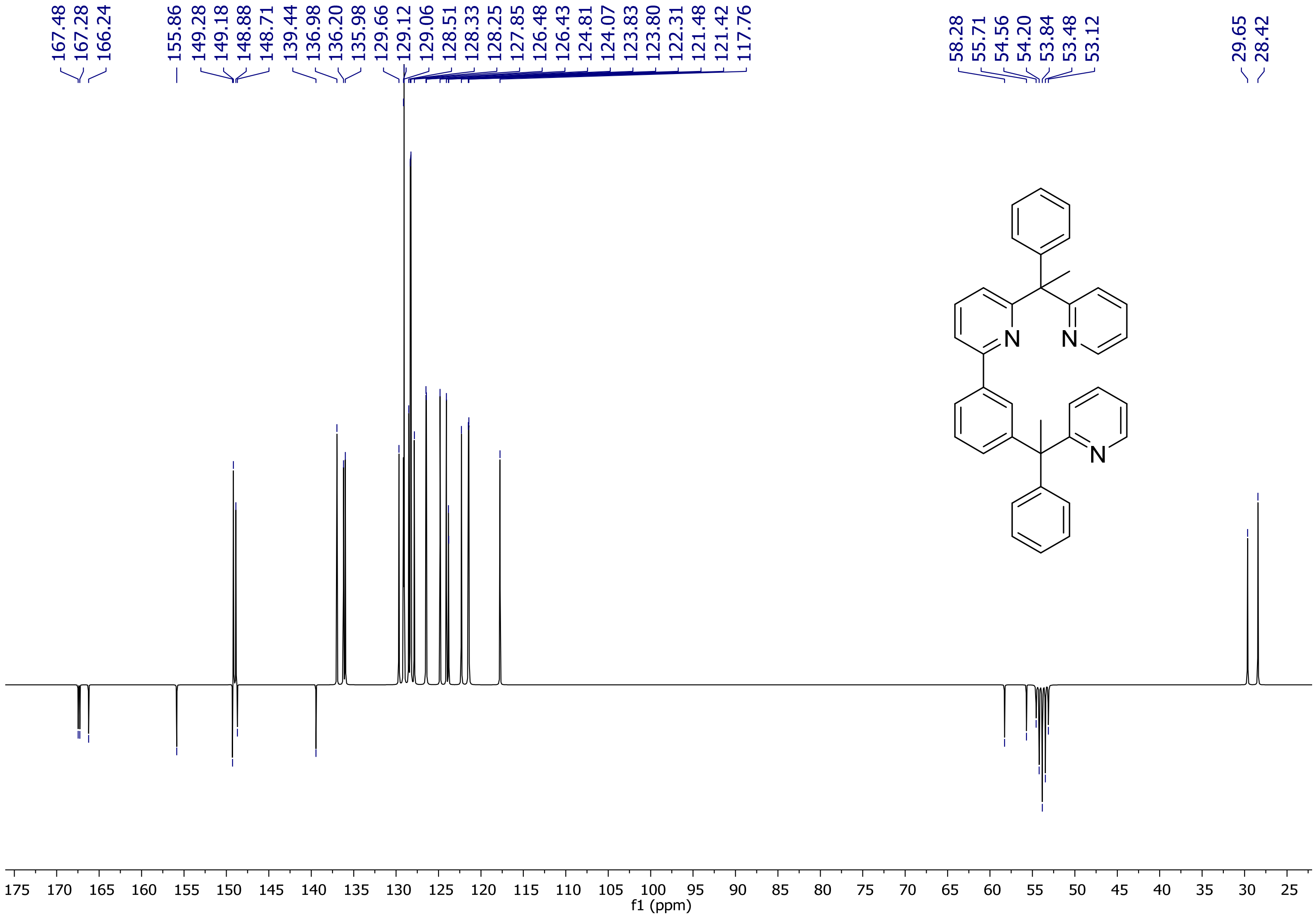

Figure S26. $^{13}C\{^{1}H\}$-APT NMR (75 MHz, CD_2Cl_2, 298 K) spectrum of **H_3L** (**7**).

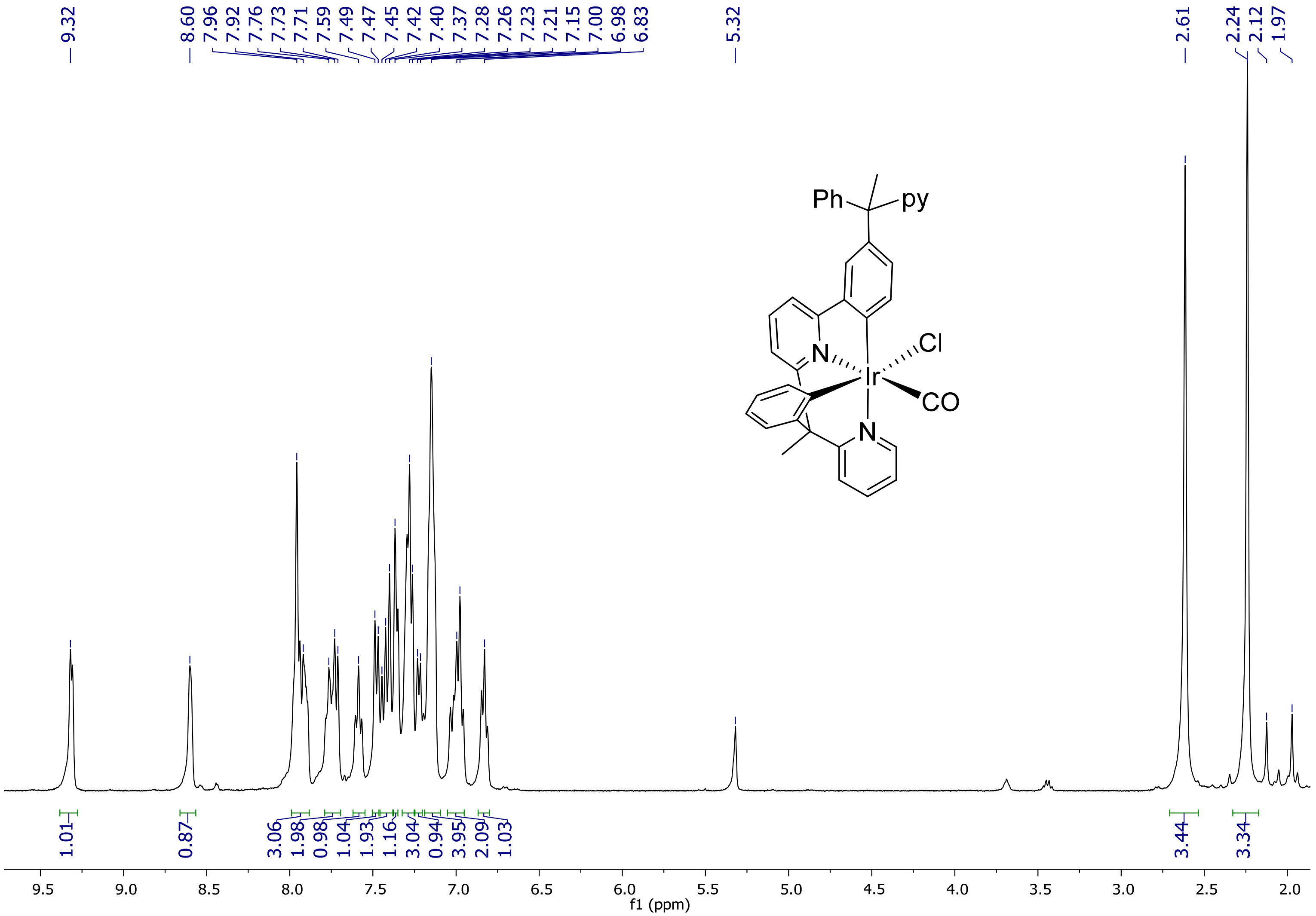

Figure S27. ^{1}H NMR (300 MHz, CD_2Cl_2, 298 K) spectrum of **9**.

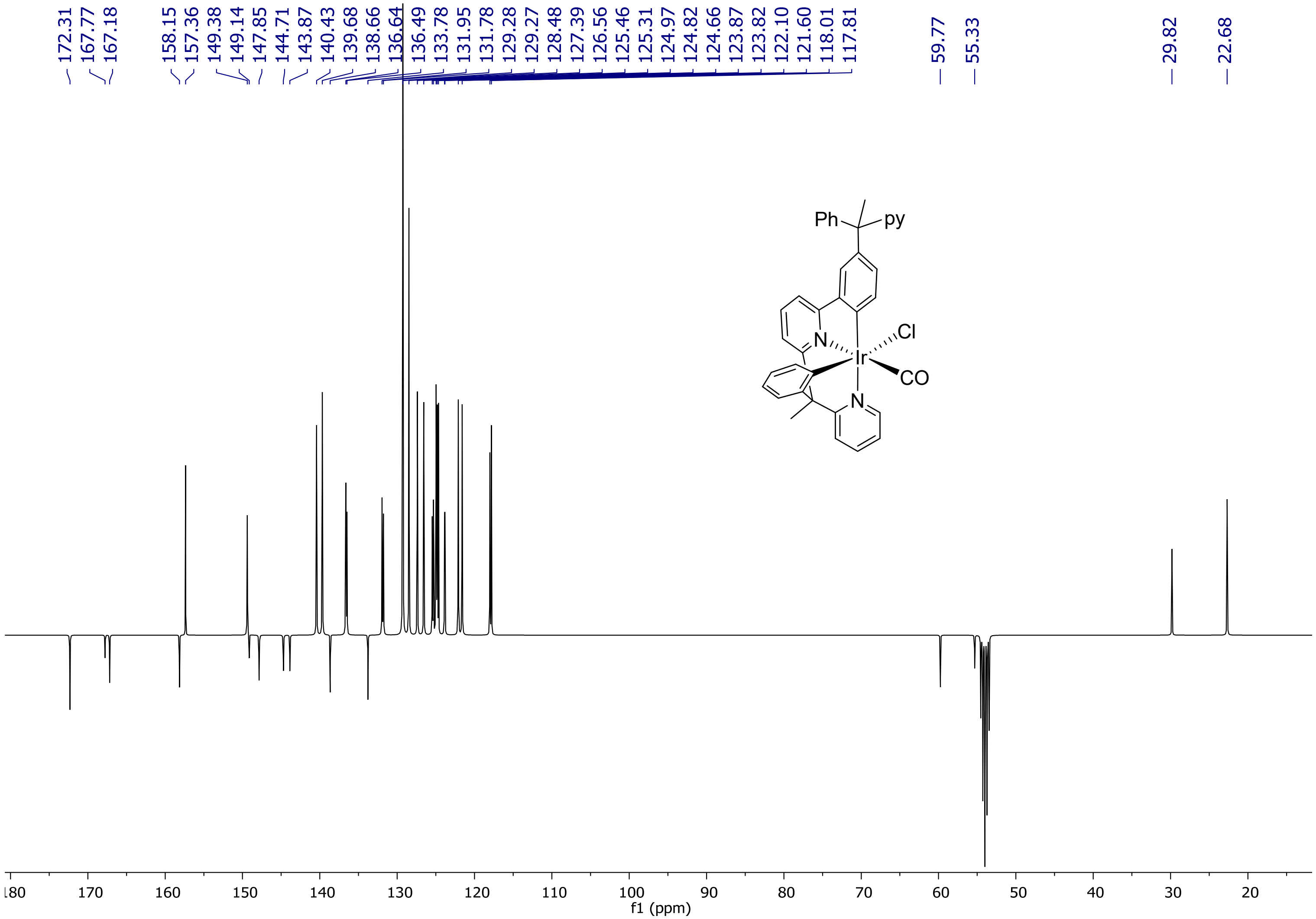

Figure S28. $^{13}C\{^{1}H\}$-APT NMR (75 MHz, CD_2Cl_2, 298 K) spectrum of complex **9.**

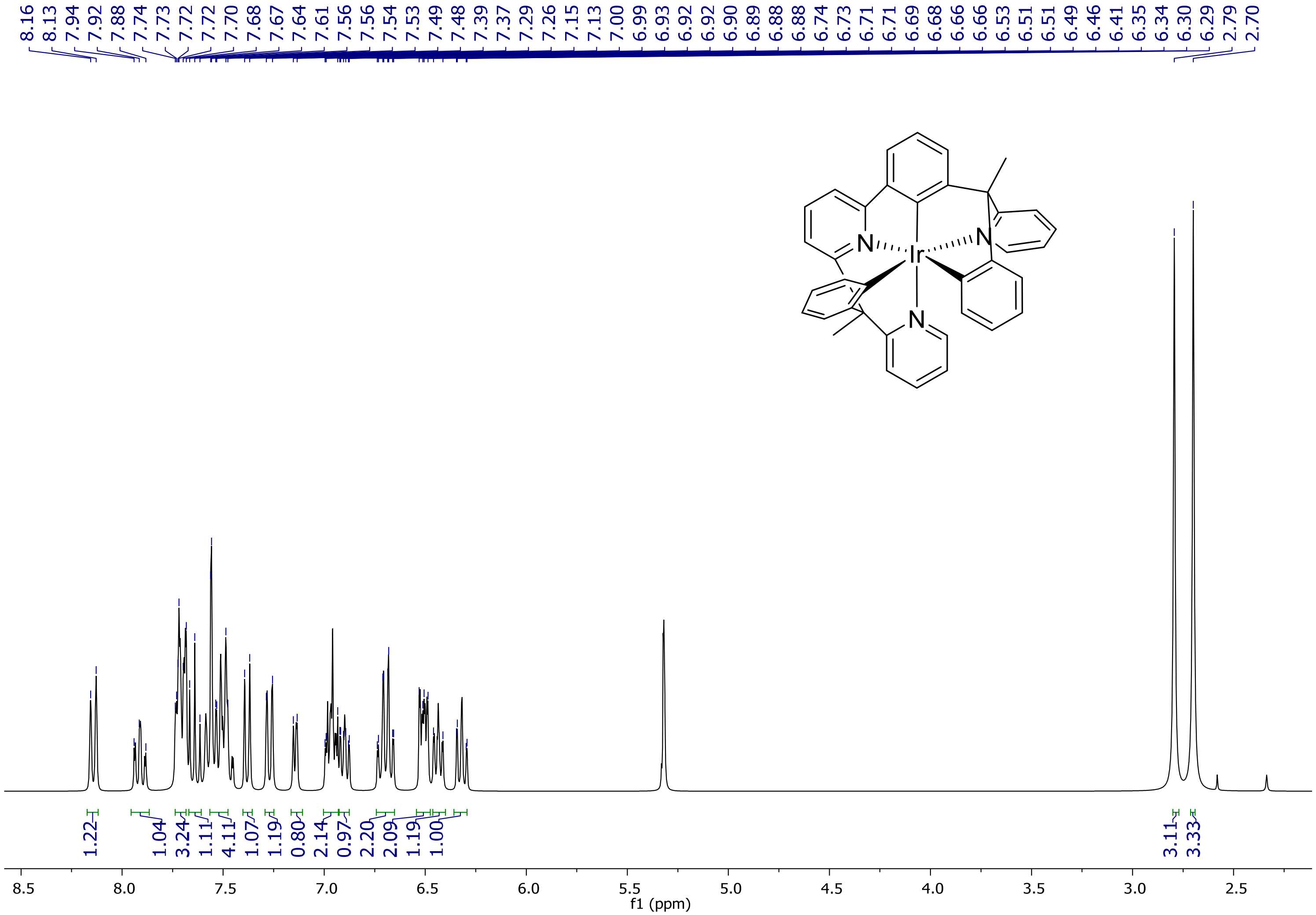

Figure S29. ^{1}H NMR (300 MHz, CD_2Cl_2, 298 K) spectrum of complex **10**.

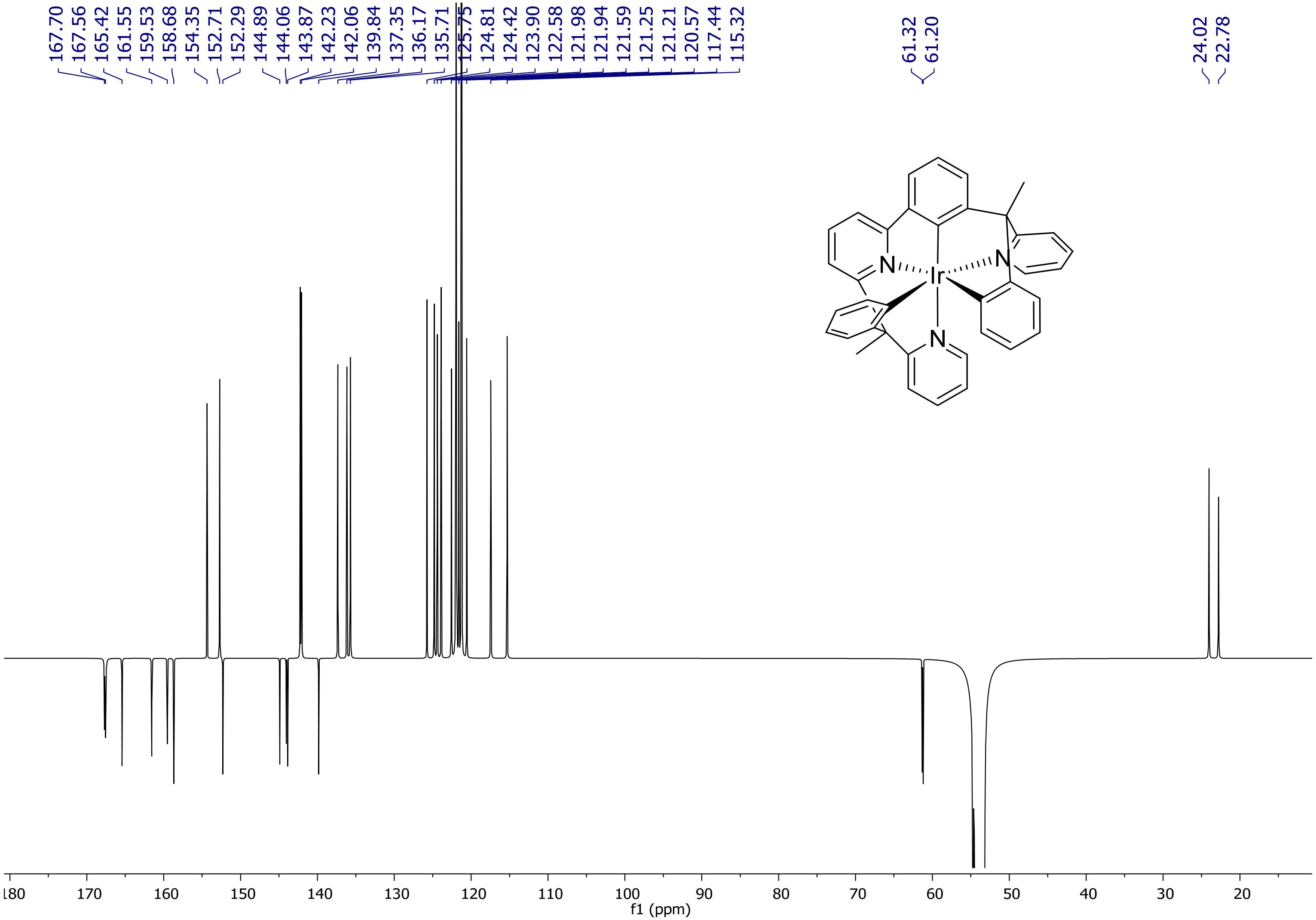

Figure S30. $^{13}C\{^{1}H\}$-APT NMR (75 MHz, CD_2Cl_2, 298 K) spectrum of complex **10**

References

(1) Blessing, R. H. An Empirical Correction for Absorption Anisotropy. *Acta Crystallogr.* **1995**, *A51*, 33. SADABS: Area-detector absorption correction; Bruker- AXS, Madison, WI, 1996.

(2) SHELXL-2016/6. Sheldrick, G. M. A short history of SHELX. *Acta Cryst.* **2008**, *A64*, 112-122.

(3) (a) Lee, C.; Yang, W.; Parr, R. G. Development of the Colle-Salvetti correlation-energy formula into a functional of the electron density. *Phys. Rev. B* **1988**, *37*, 785–789. (b) Becke, A. D. Density-functional exchange-energy approximation with correct asymptotic behavior. *J. Chem. Phys.* **1993**, *98*, 5648–5652. (c) Stephens, P. J.; Devlin, F. J.; Chabalowski, C. F.; Frisch, M. J. *Ab Initio* Calculation of Vibrational Absorption and Circular Dichroism Spectra Using Density Functional Force Fields. *J. Phys. Chem.* **1994**, *98*, 11623–11627.

(4) Grimme, S.; Antony, J.; Ehrlich, S.; Krieg, H. A consistent and accurate *ab initio* parametrization of density functional dispersion correction (DFT-D) for the 94 elements H-Pu. *J. Chem. Phys.* **2010**, *132*, 154104.

(5) Gaussian 09, Revision D.01, Frisch, M. J.; Trucks, G. W.; Schlegel H. B.; Scuseria, G. E.; Robb, M. A.; Cheeseman, J. R.; Scalmani, G.; Barone, V.; Mennucci, B.; Petersson, G. A.; Nakatsuji, H.; Caricato, M.; Li, X.; Hratchian, H. P.; Izmaylov, A. F.; Bloino, J.; Zheng, G.; Sonnenberg, J. L.; Hada, M.; Ehara, M.; Toyota, K.; Fukuda, R.; Hasegawa, J.; Ishida, M.; Nakajima, T.; Honda, Y.; Kitao, O.; Nakai, H.; Vreven, T.; Montgomery, J. A.; Peralta, Jr., J. E.; Ogliaro, F.; Bearpark, M.; Heyd, J. J.; Brothers, E.; Kudin, K. N.; Staroverov, V. N.; Keith, T.; Kobayashi, R.; Normand, J.; Raghavachari, K.; Rendell, A.; Burant, J. C.; Iyengar, S. S.; Tomasi, J.; Cossi, M.; Rega, N.; Millam, J. M.; Klene, M.; Knox, J. E.; Cross, J. B.; Bakken, V.; Adamo, C.; Jaramillo, J.; Gomperts, R.; Stratmann, R. E.; Yazyev, O.; Austin, A. J.; Cammi, R.; Pomelli, C.; Ochterski, J. W.; Martin, R. L.; Morokuma, K.; Zakrzewski, V. G.; Voth, G. A.; Salvador, P.; Dannenberg, J. J.; Dapprich, S.; Daniels, A. D.; Farkas, O.; Foresman, J. B.; Ortiz, J. V.; Cioslowski, J.; Fox, D. J. Gaussian, Inc., Wallingford CT, 2013.

(6) Andrea, D.; Häußermann, U. M.; Dolg, M.; Stoll, H.; Preuss, H. Energy-adjusted *ab initio* pseudopotentials for the second and third row transition elements. *Theor. Chim. Acta* **1990**, *77*, 123–141.

(7) Ehlers, A. W.; Bohme, M.; Dapprich, S.; Gobbi, A.; Hollwarth, A.; Jonas, V.; Kohler, K. F.; Stegmann, R.; Veldkamp, A.; Frenking, G. A set of f-polarization functions for pseudo-potential basis sets of the transition metals SC-Cu, Y-Ag and La-Au. *Chem. Phys. Lett.* **1993**, *208*, 111–114.

(8) (a) Hehre, W. J.; Ditchfield, R.; Pople, J. A. Self-Consistent Molecular Orbital Methods. XII. Further Extensions of Gaussian-Type Basis Sets for Use in Molecular Orbital Studies of Organic Molecules. *J. Chem. Phys.* **1972**, *56*, 2257–2261. (b) Francl, M. M.; Pietro, W. J.; Hehre, W. J.; Binkley, J. S.; Gordon, M. S.; DeFrees, D. J.; Pople, J. A. Self-consistent molecular orbital methods. XXIII. A polarization-type basis set for second-row elements. *J. Chem. Phys.* **1982**, *77*, 3654–3665.

(9) Marenich, A. V.; Cramer, C. J.; Truhlar, D. G. Universal Solvation Model Based on Solute Electron Density and on a Continuum Model of the Solvent Defined by the Bulk Dielectric Constant and Atomic Surface Tensions. *J. Phys. Chem. B* **2009**, *113*, 6378–6396.

(10) O'Boyle, N. M.; Tenderholt, A. L.; Langner, K. M. cclib: A Library for Package-Independent Computational Chemistry Algorithms.*J. Comput. Chem.* **2008**, *29*, 839−845.
